# Protective Action Mechanisms of *Launaea mucronata* Extract and Its Nano-Formulation against Nephrotoxicity in Rats as Revealed via Biochemical, Histopathological, and UPLC-QTOF–MS/MS Analyses

**DOI:** 10.3390/metabo13070786

**Published:** 2023-06-23

**Authors:** Amany A. El-Fadaly, Inas Y. Younis, Mohamed F. Abdelhameed, Yasmine H. Ahmed, Tamer I. M. Ragab, Abd El-Nasser G. El Gendy, Mohamed A. Farag, Abdelsamed I. Elshamy, Abdelbaset M. Elgamal

**Affiliations:** 1 Pharmacology Department, National Research Centre, 33 El Bohouth St., Dokki, Giza 12622, Egypt; aa.elfadaly@nrc.sci.eg (A.A.E.-F.); mf.abdelhameed@nrc.sci.eg (M.F.A.); 2 Pharmacognosy Department, Faculty of Pharmacy, Cairo University, Kasr el Aini St., Cairo 11562, Egypt; inas.younis@pharma.cu.edu.eg (I.Y.Y.); mohamed.farag@pharma.cu.edu.eg (M.A.F.); 3 Department of Cytology and Histology, Faculty of Veterinary Medicine, Cairo University, Giza 12211, Egypt; yasmine_hamdi@cu.edu.eg; 4 Chemistry of Natural and Microbial Products Department, National Research Centre, 33 El-Bohouth St., Dokki, Giza 12622, Egypt; ti.ragab@nrc.sci.eg; 5 Medicinal and Aromatic Plants Research Department, National Research Centre, Dokki, Giza 12622, Egypt; ag.el-gendy@nrc.sci.eg; 6 Chemistry of Natural Compounds Department, National Research Centre, 33 El Bohouth St., Dokki, Giza 12622, Egypt

**Keywords:** *Launaea mucronata*, secondary metabolites, renal injury, potassium dichromate toxicity, oxidative stress, UPLC-MS

## Abstract

Plants belonging to the *Launaea* genus have been extensively utilized ethnopharmacologically to treat a variety of diseases, including kidney disorders. Chromium is a common industrial pollutant that has been linked to kidney disease. The present work was designed for the investigation of the UPLC-QTOF–MS/MS metabolite profile of the *L. mucronate* ethanolic extract (LME), along with assessing the mechanistic protective actions of LME and its nano-silver formulation (LMNS) against K_2_Cr_2_O_7_-induced nephrotoxicity in rats. LMNE was successfully biosynthesized and confirmed using UV–Visible (UV–Vis) spectroscopy and transmission electron microscopy (TEM). The nephroprotective effects of LME and LMNE was assessed in rats exposed to potassium dichromate (K_2_Cr_2_O_7_, 15 mg/kg BW) to cause nephrotoxicity. LME and LMNS, separately, were administered twice daily for 14 days at doses of 200 and 400 mg/kg BW, respectively. The kidney function, catalase, UGT, Nrf2, PGE2, Cox-2, ERK, and MAPK levels in renal tissue were all assessed, along with histopathological examinations for exploring their ameliorative effects. Forty-five bioactive metabolites were annotated belonging to flavonoids, phenolic and organic acids, coumarins, and fatty acids. Metabolite profiling revealed that chlorogenic acid, apigenin, and luteolin glycosides were the main phenolics, with chlorogenic acid-*O*-hexoside reported for the first time in LME. The findings revealed that the serum kidney function indicators (urea and creatinine) were markedly elevated in K_2_Cr_2_O_7_-intoxicated rats. Furthermore, inflammatory indicators (COX-2 and PGE2), MAPK, and ERK were all markedly elevated in kidney tissue, whereas catalase, UGT, and Nrf2 levels were downregulated. Histological and immunohistochemical assays confirmed the toxic effects of K_2_Cr_2_O_7_ in the kidneys. In contrast, the administration of LME and LMNS prior to K_2_Cr_2_O_7_ considerably improved the architecture of the renal tissue, while also restoring levels of most biochemical markers. Functioning via the inhibition of the MAPK/ERK pathway, activating Nrf2, and modifying the antioxidant and metabolic enzymes, LME and LMNS exerted their nephroprotective effects against K_2_Cr_2_O_7_-induced toxicity.

## 1. Introduction

Medicinal plants and their bioactive by-products are well recognized for their several medicinal and pharmaceutical applications [1]. *Launaea* Cass. (Family Asteraceae), including around 54 species, is a common genus around the world, especially in Africa, the South Mediterranean, and Asia [1,2]. Several traditional uses were reported for *Launaea* plants worldwide, including the treatment of the ailments of stomach, breast, hepatic, skin, and insect infections, alongside of inflammation, wounds, diarrhea, fever, and gastrointestinal diseases [3,4,5,6]. Furthermore, bioassays confirmed many of *Launaea* species extracts and/or their isolated chemicals, including antioxidant, antidiabetic, insecticidal, anticancer, antifungal, anti-inflammatory, anti-angiogenic, and antimicrobial activity [1,3,4,5,6,7,8]. Phytochemical studies revealed several bioactive metabolites from the different extracts of LME, including flavonoids, coumarins, sesquiterpenes [9], and essential oil [1] to account for its health benefits.

The kidney is the primary vital organ that carries out various crucial functions, such as detoxification, extracellular fluid management, homeostasis, and the excretion of harmful compounds [10,11]. Nephrotoxicity is defined as a sharp decline in kidney functioning induced by the toxic effects of drugs and chemical substances [10,12].

Hexavalent chromium (CrVI) has been used in stainless steel manufacturing, leather tanning, and wood preservation, and was likewise detected in drinking water, thereby posing as a potential contaminant [13]. It can enter cells and trigger oxidative stress leading to a variety of issues, including skin rashes, allergic reactions, immune system deterioration, irritations and bleeding of the nose, genetic material alteration, kidney and liver damage, and even death for the person [14]. The kidney is the primary target for Cr excretion. According to a previous study, rats given an acute dose of potassium dichromate had higher levels of Cr in their kidneys. Cr (VI) compound exposure can induce nephrotoxicity in humans and experimental animals mediated via the generation of reactive oxygen species (ROS) concurrent with a decrease in antioxidant enzyme activity, as well as a reduction in the renal blood flow, perfusion, and oxygenation levels [15].

The creation of nano-formulations of plant extracts has an enormous potential in the field of nanomedicine, including boosting their biological effects through augmentation of the active biomolecule’s concentration [16]. Due to their distinctive applications in pharmaceutics, agriculture, water detoxification, air filtration, textile industries, and as a catalyst, silver nanoparticles have received increasing attention among these nano-formulations [17,18,19].

The loading of bioactive constituents in nano-systems represents one of the most significant modern techniques for the development of medicinal drugs via several pathways, including (1) the enhancement of the bioactivities of the targeting products, (i) decreased toxicity, (ii) reducing volatility, (iii) increasing stability of the active components, and (iv) increasing the penetration inside the tissues and cellular uptake. Additionally, the creation of nano-formulations of plant extracts has an enormous potential in the field of nano-medicine, including boosting their biological effects through augmentation of the active biomolecules [17,18,19].

The objectives of present work were to: (i) investigate the chemical profile of LME using UPLC-QTOF-MS/MS analysis as a platform for extract profiling in untargeted manner; (ii) bio-synthesize and characterize the silver nano-formulation of *L. mucronata* hydroethanolic extract (LMNS); and (iii) assess the protective mechanisms of LME and LMNS against K_2_Cr_2_O_7_-induced nephrotoxicity in rats via biochemical and histochemical assays.

## 2. Materials and Methods

### 2.1. Plant Material and Collection

The collection and authentication of *L. mucronata* aerial parts was conducted by Prof. Ahmed Abdel Gawad, Prof. of taxonomy, Mansoura University from the Wadi Hagul, (30°02′34.3′′ N, 32°05′40.6′′ E); El-Kattamyia-El-Ain Sokhna Road, Cairo, Egypt. The collection was performed in the early morning (around 4.5 to 6.5 AM) of Monday 26 April 2021. The collected plant material was assigned a voucher number (LM-yM-8741x/21-06623) and was deposited at the Herbarium of Faculty of Science, Mansoura University. *L. mucronata* aerial parts were cleaned, and dried completely in an open, shaded room at a room temperature (25–28 °C) for 10 days, and then ground into a powder.

### 2.2. Extract Preparation

A 70% EtOH (3 L) extraction was performed on 870 g of air-dried powdered plant aerial parts for 5 days and then filtered. This extraction was conducted three times successively, following which the solvent portions were collected, and dried under reduced pressure at 45 °C to yield a dark gum (28.6 g), which was kept at 4 °C until further chemical and biological assays.

### 2.3. High-Resolution Ultra-Performance Liquid Chromatography-Mass Spectrometry Analysis (UPLC-qTOF-MS)

For the profiling of LME using UPLC-TOF-MS, 1 g was extracted using 70% EtOH in an ultrasonic bath (Branson ultrasonic corporation, Danbury, CT, USA) for 1 h. After filtering and centrifugation for 15 min at 12,000× *g*, the clear supernatant was used for UPLC-qTOF-MS analysis. The UPLC-qTOF-MS analysis of the extract was conducted under the exact conditions reported in [10,18]. The used HSS T3 column (100 × 1.0 mm, 1.8 m particle size; Waters) was installed on an ACQUITY UPLC system (Waters, Milford, MA, USA) equipped with a 6540 Ultra-High-Definition (UHD) Accurate-Mass Q-TOFLC/MS (Agilent, Palo Alto, CA, USA), coupled to an ESI interface, and was operated in a positive or negative ion mode.

### 2.4. Biosynthesis of the Silver Nanoparticles of LMNS

A stock aqueous 10 mL AgNO_3_ solution at 25 °C and various concentrations (100–500 µL) of LME were added to this reaction mixture (1 mM). The reaction mixture was shaken slightly and allowed to stand in the dark at room temperature overnight, and then filtered with the filter paper Whatman 1 [20]. Ag NPs were produced when AgNO_3_ solution was reduced with LME.

### 2.5. UV–Vis Spectra and Transmission Electron Microscopy (TEM) Measurements of LMNS

The absorbance spectra of nanoparticle solutions were recorded following the synthesis (within 1 h) using de-ionized water as the blank. A Shimadzu UV-2401 (PC) S, UV-Vis spectrophotometer was used and operated using Spectrum TM Version 6.87 (Shimadzu, Japan) scanning from 200–800 nm. The maximum absorption wavelength, λ_max_ was then noted [18]. To assess the particle size and shape, a JEOL JEM 1011 (JEOL Ltd., Tokyo, Japan) transmission electron microscope was employed. Then, 400 μL of nanoparticle solutions were applied to copper grids (400 mesh) that had been coated with carbon and dried at 30 °C prior to image capturing [21].

### 2.6. Bioassays

#### 2.6.1. Drugs and Chemicals

Potassium dichromate (K_2_Cr_2_O_7_) [CAS# 7778-50-9] was purchased from Sigma-Aldrich Chemical Co. St. Louis, Missouri, (USA). Ethanol (96%; CAS #: 64-17-5) was purchased from Merck Millipore (MS, USA). Creatinine (cat# CR 12 51), Urea (cat# UR 21 10) and catalase (cat# CA 25 17) colorimetric kits were both purchased from Bio Diagnostic (Giza, Egypt), while the ELISA kits, including nuclear factor erythroid 2-related factor 2 (Nrf2, cat# SL0985Ra), prostaglandin E2 (PGE2, cat# SL0601Ra), cyclooxygenase 2 (COX-2, cat# SL0218Ra), UDP-glucuronosyltransferase (cat# SL1171Ra), mitogen-activated protein kinase (MAPK) (cat# SL1529Ra), and extracellular signal-regulated kinase (ERK) (cat# SL1390Ra), were all obtained from (SUNLONG BIOTECH CO., Ltd.), Hangzhou, China.

#### 2.6.2. Acute Toxicity

As according to the procedures outlined by previous reports [22], 2-month-old male and female Swiss albino mice with an average weight of 20.4 g were used to assess the toxicity of the LME and its LMNS applying the OECD guideline no. 420. The mice were separated into 4 groups consisting of 5 animals each, who were acclimated for 5 days prior to the test experiments, and then starved overnight before dosing. The mice were given a single dose of the tested material (2000 mg/kg) by gavage, and their ears, skin, mucous membranes, eyes, respiration, circulatory, autonomic, and somatomotor activities were observed for any alterations. In particular, convulsions, tremors, diarrhea, salivation, lethargy, sleep, and coma, behavior patterns were all observed. The extract under test did not exhibit any fatalities or toxic indications up to an extract dose of 2000 mg/kg body weight, indicating that it is non-toxic and safe to use.

#### 2.6.3. Experimental Animals and Ethical Treatments

Around 48 male Albino Wister rats (with an average weight of 140–150 g) were purchased from the Animal Facility of the National Research Centre, Egypt. The animals were kept in standard cages under pathogen-free conditions, maintained in an environmentally controlled room (at 22–25 °C, and 50–60% humidity with a 12 h light/dark cycle,) and received a standard laboratory diet and water ad libitum. The rats were allowed to adapt to these conditions for 2 weeks prior to beginning the experimental protocol. All studies were conducted in accordance with the Cairo University’s Ethical Committee’s [Approval No: Vet CU 03162023753] authorized Ethical Guidelines for the Care and Use of Experimental Animals.

#### 2.6.4. Experimental Design

Animals were randomly allocated into six groups (*n* = 8), which are as follows: group 1: normal control (healthy normal control), wherein rats received normal saline solution (0.9%) for 14 days, group 2: (positive control), whereby the selected rats received K_2_Cr_2_O_7_ (15 mg/kg body weight; i.p) once on day 13 of the experiment [15], groups 3 and 4, wherein rats received LME (200 and 400 mg/kg body weight; p.o, respectively) daily for 14 days, and groups 5 and 6, wherein rats received LMNS (200 and 400 mg/kg body weight; p.o, respectively) daily for 14 days. Nephrotoxicity was induced by a single dose of 15 mg/kg BW i.p. injection of K_2_Cr_2_O_7_ to all groups except for the normal control group on day 13 of the experiment [23] (Figure S1).

#### 2.6.5. Blood Collection and Tissue Preparation

Forty-eight hours after the last treatment, rats were anaesthetized for blood sample collection from the retro-orbital plexus. Blood was first collected in clean centrifuge tubes, left to clot, and then centrifuged for 10 min at 1409× *g* using a cooling centrifuge (Sigma and Laborzentrifugen, 2 k15, Germany). The serum was separated and stored in Eppendorf tubes at −80 °C to be used for the assessment of the creatinine and urea levels. Kidneys were then carefully dissected and thoroughly cleansed with PBS buffer after all animals were quickly euthanized by cervical dislocation. For histological analysis, a portion of the kidney tissues of a predetermined number of animals in each group were fixed in 10% formalin buffer for 24 h, while the remaining kidney tissues (0.5 g) were homogenized in 10% (*w*/*v*) phosphate buffer, which was ice cold. At 4 °C for 10 min, the homogenate was centrifuged at 1800× *g*. The supernatant was put into Eppendorf tubes and kept at a low temperature to be used for the measuring of the biochemical parameters.

#### 2.6.6. Assays for Kidney Function

The serum urea and creatinine were determined using colorimetric kits according to the manufacturer’s instructions (Bio Diagnostic, Giza, Egypt) [24].

#### 2.6.7. Biochemical Assessment of Renal Tissue

Catalase activity was assessed using the colorimetric method, while the levels of nuclear factor erythroid 2-related factor 2 (Nrf2), prostaglandin E2 (PGE2), cyclooxygenase 2 (COX-2), UDP-glucuronosyltransferase, mitogen-activated protein kinase (MAPK), and extracellular signal-regulated kinase (ERK) were all assessed in renal homogenates using ELISA kits following the manufacturer’s instructions.

#### 2.6.8. Histopathological Assays

##### Light Microscopic Examination

Fixed kidney samples were dehydrated with a series of 100% alcohol washes followed by xylene and embedded in paraffin. Sections comprising 4 μm thick were prepared using a rotatory microtome, deparaffinized, and stained with hematoxylin and eosin (H&E) for histopathological examination [25].

##### Immunohistochemical Examination

The presence of the cyclooxygenase 2 protein (COX 2), a dark, brown-colored stained cytoplasm, was considered as a positive response. According to Cote, 1993 [26], the used methods were (i) image analysis to assess immunohistochemical observations (area percentage), and (ii) a digital Leica Quin 500Â image analysis system (Leica Microsystems, Switzerland) housed at the Faculty of Dentistry, Cairo University for the analysis of sections stained with anti-COX-2 (Catalogue No.: PA1-37504, ThermoFisher Scientific), Waltham, Massachusetts, USA. 

#### 2.6.9. Statistical Analysis

All results were expressed as mean ± SD. Data analysis was achieved by the one-way analysis of variance (ANOVA, to determine the significance of the mean between the groups) followed by the Tukey’s multiple comparison test. *p*-value < 0.05 was considered statistically significant using the software program GraphPad Prism (version 7.00; GraphPad Software, Inc., San Diego, CA, USA). 

## 3. Results

### 3.1. LME’ Phytochemical Profiling Using UPLC-QTOF–MS/MS

**Figure 1:**
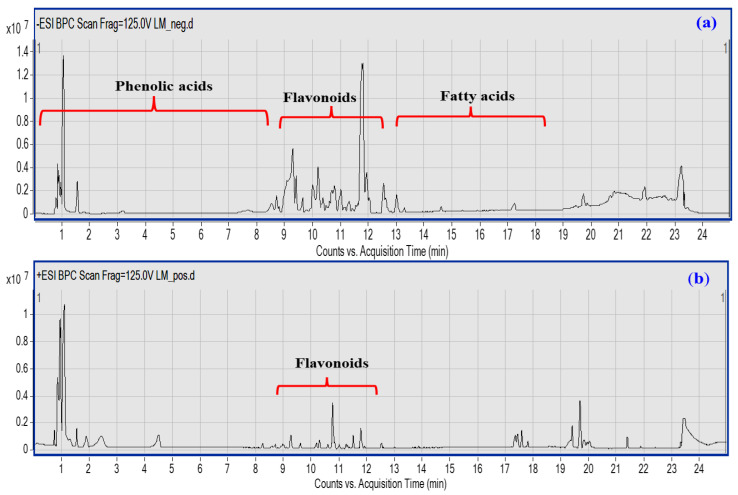
UPLC-qTOF-MS base peak chromatogram of LME metabolites detected in their (**a**) negative and (**b**) positive ionization modes. Peak numbers follow of that listed in Table 1 for the identified metabolites.

The UPLC-QTOF–MS/MS-based metabolic profiling of LME is a powerful tool for the characterization of natural bioactive metabolites in plant extracts at a high sensitivity level [27]. A total of 45 compounds were putatively identified belonging to various classes, *viz*; phenolic acids, flavonoids, coumarins, and fatty acids based on their characteristic fragmentation patterns as acquired in both negative and positive ionization modes (Figure 1a,b), in addition to the previously reported literature. Details of each class identification are provided in the next subsections.

### 3.2. Identification of Hydroxycinnamic and Hydroxy Benzoic Acids

Hydroxycinnamic (HCAs) and hydroxy benzoic acids are natural phenolic compounds with an excellent antioxidant activity. Structurally, the C6-C3 phenylpropanoid skeleton is the primary scaffold of HCAs that are recognized by the attachment of hydroxyl group(s) to an aromatic ring and the carboxyl group present in the lateral chain. The substitution and the position of these hydroxyl groups contribute to the diversity of the HCAs [28]. In the current study, both cinnamates and benzoates were detected either in their free form, esterified with quinic or tartaric acids, or bound to sugar moieties, as depicted in Figure 1 and Table 1.

Delving into further detail, peaks 7 and 18 displayed [M-H]^−^ at *m*/*z* 341.00878 (C_15_H_17_O_9_^−^) and 515.0831 (C_24_H_19_O_13_^−^), respectively, accompanied with a characteristic loss of the hexose unit (162 amu) along with the generation of their corresponding acids at *m*/*z* 179 and 353, respectively (Otify 23). Both were annotated as caffeoyl-*O*-hexose and chlorogenic acid-*O*-hexoside, respectively (Figures S2 and S3). The former was previously identified in *L. nudicaulis* aerial parts [8], whereas chlorogenic acid-*O*-hexoside was identified for the first time in LME. Likewise, peak 8 (315.0722, C_13_H_15_O_9_^−^) showed an intense ion peak of protocatechuic acid after the elimination of the hexose unit (Figure S4) [29].

#### 3.2.1. Identification of Coumarins

Coumarins are defined as natural bioactive molecules containing a benzopyranone core. They have demonstrated diverse pharmacological activities *viz*; anti-inflammatory, anticoagulant, antimicrobial, and anti-Alzheimer effects [30]. Indeed, at peaks 14 and 15, esculetin (*m*/*z* 177.019, C_9_H_5_O_4_^−^) and its glycoside esculin (*m*/*z* 339.0722, C_15_H_15_O_9_^−^) were characterized by the presence of the diagnostic fragment at *m*/*z* 177 post-elimination of hexose (Figures S5 and S6). Esculetin was previously isolated from *L. spinosa* [31], while, esculin was the first time to be reported in LME.

#### 3.2.2. Identification of Flavonoids

Flavonoids are natural secondary metabolites containing the main structural unit of 2-phenylchromone. They are ubiquitously distributed across various vegetables and fruits, with luteolin and apigenin being the most abundant flavones detected in their glycosylated forms. As illustrated in Table 1, 8 luteolin and apigenin glycosides were identified along with their parent aglycones. In the MS/MS spectra, peaks 21 (461.0725, C_21_H_17_O_12_^−^) and 22 (447.0933,C_21_H_19_O_11_^−^) displayed a diagnostic fragment ion at *m*/*z* 285 due to the successive loss of the glucuronic acid (−176 amu) (Figure S7) and hexose units (−162 amu) (Figure S8), with the liberation of luteolin aglycone [32]. Therefore, they were assigned as luteolin-3-*O*-glucouronic acid and luteolin-3-*O*-hexoside, respectively. Higher intensity of fragment ions observed at *m*/*z* 285 [M-H-162]^−^ than 284 [M-H-162]^−^ was deemed to be indicative of the heterolytic cleavage and the glycosylation site at 3-OH (Figure S8). Luteolin can be distinguished from kaempferol by the presence of a series of diagnostic fragments at *m*/*z* 175, 151, and 133, respectively (Figure S9) [33]. A similar fragmentation pattern was observed in peaks 27 and 29, which were identified as apigenin-3-*O*-hexoside and apigenin-3-*O*-glucouronic acid, respectively, with the elimination of [M-H-162]^−^ and [M-H-176]^−^ yielding apigenin aglycone at *m*/*z* 271 (Figure S10) [34].

#### 3.2.3. Identification of Fatty and Organic Acids

Linoleic acid is an essential polyunsaturated fatty acid (FA) that plays a crucial role in reducing the incidence of heart diseases and decreasing cardiometabolic biomarkers, such as LDL [35]. In the same context, palmitic acid (16:0) is one of the most important saturated FA that constitutes 20–30% of the total FA in the phospholipid bilayer membranes [36]. Notably, hydroxylated linoleic and palmitic acids (at peaks 44 and 45, respectively) displayed diagnostic fragment ions formed through the loss of water, CO_2_, or both moieties [8]. The esters of fatty acids have evoked increasing interests due to their potential anti-inflammatory activity [37].

In regard to the organic acids, four acids were identified based on the characteristic MS^2^ spectrum and displayed intense precursor peaks at *m*/*z* 191, 133, 167, 290, 187, and 227 for quinic, malic acid, tartaric acid, deoxy-dehydro-*N*-acetylneuraminic acid, nonanedioic acid, and hydroxy jasmonic acid, respectively. These acids were previously detected in *L. nudicaulis* alcoholic extract [8].

### 3.3. Chemical Biosynthesis of LMNS

#### 3.3.1. UV–Vis Spectroscopic Analysis of LMNS

**Figure 2:**
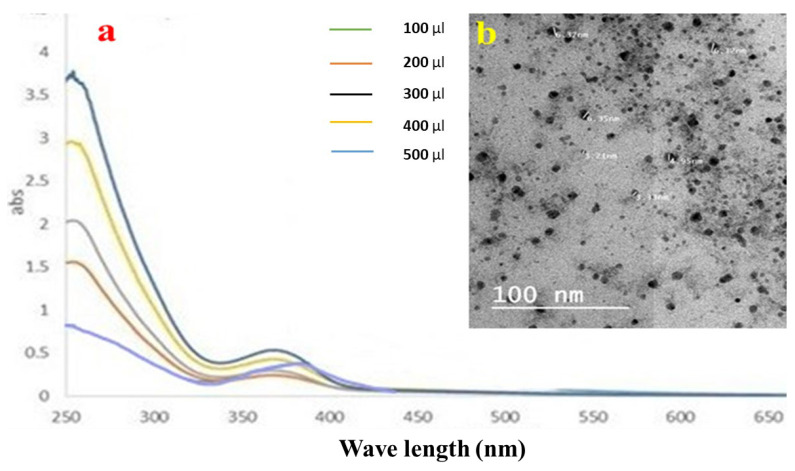
(**a**) UV–Vis spectroscopic data, and (**b**) the TEM image of the silver nanoparticle distribution of LME.

A plasmon band between 445 and 456 nm, which is typical of silver nanoparticles, was visible in the samples’ UV–Vis spectra [38]. The Ag NPs solutions vary in color from colorless to pale yellow to dark brown, depending on the number of nanoparticles produced. Even after 30 min, the hue remained constant, thereby showing that there was no obvious particle aggregation. The aggregation was then indicated with a color shift to darkness, and a clear solution with precipitated black silver was evident. Ag NPs were produced as a result of reducing the AgNO_3_ solution with LME. The absorption band emerged at longer wavelengths when using small spherical LMNS, as shown in Figure 2a. The surface plasmon resonance (SPR) band strength increased with the sample concentration, indicating that more Ag^+^ ions transformed into Ag nanoparticles [20].

#### 3.3.2. TEM Results of LMNS

The produced LMNS’s size and shape were evaluated using the HRTEM method. According to Figure 2b, the majority of the LMNS’s were spherical, with particle sizes ranging from 6.37 to 3.21 nm, respectively. Ag NPs obtained from the LME was spherical in shape with particle sizes ranging from 22.76–57.24 nm, respectively. Due to the capping effect of the plant extract during the preparation process, the particles were subsequently separated from one another [18].

### 3.4. Biological Results

#### 3.4.1. LD_50_ Assay’ Result

Swiss albino mice were administered LME and LMNS orally at doses up to 2000 mg/kg, although neither of these materials caused any visible toxicity or mortality within 24 h. The median lethal dose (LD_50_) of LME and LMNS in mice may therefore be greater than 2000 mg/kg. Compounds with LD_50_ values greater than 50 mg/kg body weight were regarded as nontoxic [39].

The general condition of animals subjected during the experiment seemed to be well during the adaptation and protective period, as no significant detected weight gain/loss was observed except for after K_2_Cr_2_O_7_ i.p. injection. The observed symptoms in animals who received potassium dichromate included several sicknesses, dullness, and ascites, and 1–2 cases of death were recorded per group. Meanwhile, all the protected groups also seemed to be generally good with no mortality observed, even for the group which received LMNS at dose of 400 mg/kg.

#### 3.4.2. Effect on Urea and Creatinine

**Figure 3:**
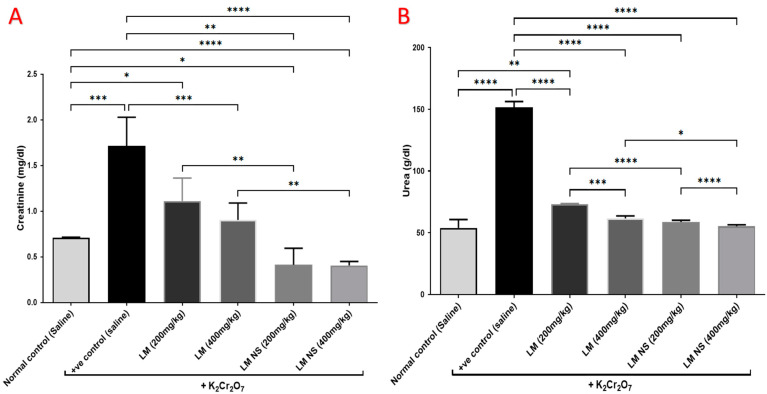
Effect of treatment with LME and LMNS on the serum (**A**) creatinine and (**B**) urea levels under nephrotoxicity induced by a single intraperitoneal injection of K_2_Cr_2_O_7_ in rats. Data are expressed as (mean ± SD) where *n* = 6. Statistical analysis was performed using the one-way analysis of variance (ANOVA) followed by the Tukey’s multiple comparison test. * *p* ≤ 0.05, ** *p* ≤ 0.01, *** *p* ≤ 0.001, **** *p* ≤ 0.0001.

Intraperitoneal injections of K_2_Cr_2_O_7_ caused a significant increase in the serum levels of urea and creatinine compared to the normal control group, thereby suggestive for its negative effects. Meanwhile, the administration of LME (at 200 and 400 mg/kg, respectively) or LMNS (at 200 and 400 mg/kg, respectively) markedly (*p* < 0.05) decreased urea and creatinine levels compared to the positive control group in a dose-dependent manner. Of note, a significant difference was observed between LME and their nanoform in decreasing urea and creatinine levels (Figure 3), with LMNS showing a better effect than LME in reducing the serum levels of urea and creatinine.

#### 3.4.3. Effect on Catalyzing Enzymes (Catalase and UGTs) and Nrf2

**Figure 4:**
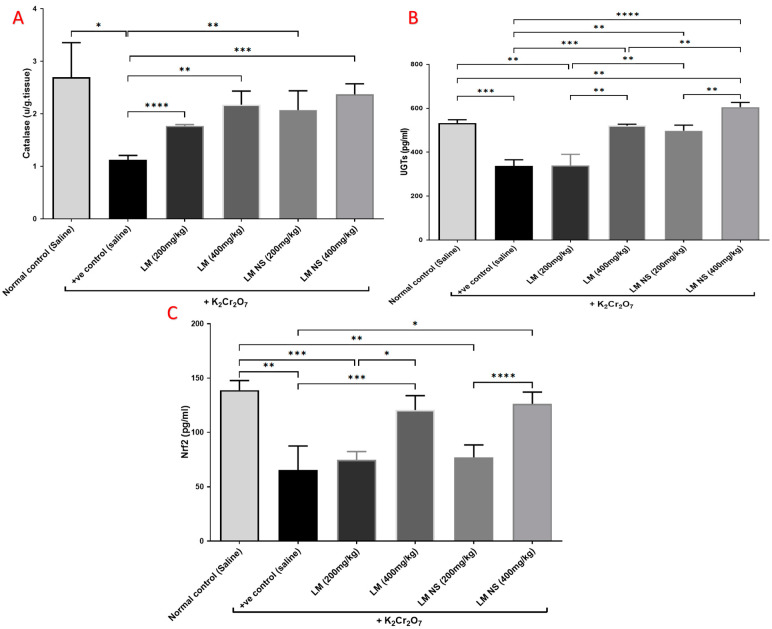
Effect of treatment with LME and LMNS on kidney (**A**) catalase, (**B**) UGT, and (**C**) Nrf2 levels in nephrotoxicity induced by a single i.p. of K_2_Cr_2_O_7_ in rats. Data are expressed as (mean ± SD) where *n* = 6. Statistical analysis was performed using the one-way analysis of variance (ANOVA) followed by the Tukey’s multiple comparison test. * *p* ≤ 0.05, ** *p* ≤ 0.01, *** *p* ≤ 0.001, **** *p* ≤ 0.0001.

Single intraperitoneal injections of K_2_Cr_2_O_7_ induced marked reductions in the levels of catalase and UGTs in kidney tissue compared to the normal control group. Meanwhile, pretreatment with LME (200 and 400 mg/kg, respectively) or LMNS (200 and 400 mg/kg, respectively) markedly (*p* < 0.05) alleviated the reduction in catalase and UGT levels compared with the positive control group in a dose-dependent manner (Figure 4a,b). There was no significant difference observed between the LME and LMNS in restoring renal catalase levels, while LMNS exerted a more potent effect than LME in restoring renal UGT levels.

Kidney content Nrf2 markedly decreased following K_2_Cr_2_O_7_ i.p. injection compared to the negative control group. Meanwhile, the administration of LME (200 and 400 mg/kg, respectively) or LMNS (200 and 400 mg/kg, respectively) caused a significant (*p* < 0.05) elevation in Nrf2 levels in the kidney as compared with the positive control group in a dose-dependent manner (Figure 4c). Furthermore, there was no significant difference observed between the LME and LMNS in increasing the renal Nrf2 levels.

#### 3.4.4. Effect on Inflammation Biomarkers (COX-2 and PGE2)

**Figure 5:**
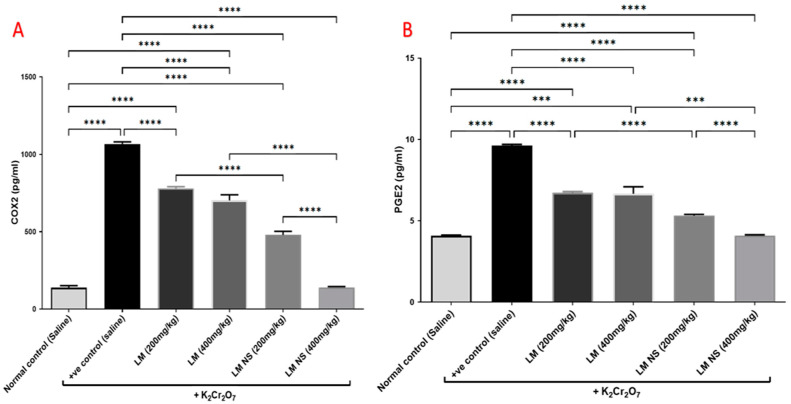
Effect of treatment with LME and LMNS on (**A**) COX-2 and (**B**) PGE2 levels in the kidney tissue under nephrotoxicity induced by a single intraperitoneal injection of K_2_Cr_2_O_7_- in rats. Data are expressed as (mean ± SD) where *n* = 6. Statistical analysis was performed using the one-way analysis of variance (ANOVA) followed by the Tukey’s multiple comparison test. * *p* ≤ 0.05, ** *p* ≤ 0.01, *** *p* ≤ 0.001, **** *p* ≤ 0.0001.

Intraperitoneal injections of K_2_Cr_2_O_7_ markedly increased renal COX-2 activity compared to the negative control group. In contrast, the administration of LMNS (200 and 400 mg/kg, respectively) significantly (*p* < 0.05) decreased COX-2 activities to the basal level compared to the positive control group. The suppression of COX-2 with LME (200 and 400 mg/kg, respectively) was achieved as a significant change when compared to the positive control group in a dose-dependent manner. Significant differences were observed between all the treatment groups where the basal level was only achieved by the LMNS (400 mg/kg) treatment (Figure 5a).

Consequently, K_2_Cr_2_O_7_ intoxication resulted in an increased production of the inflammatory mediator, PGE2 by about 1.6-fold compared to the negative control group. This finding was deemed to be probably due to increased synthesis by the actions of the COX-2 enzyme. All treatments induced a significant decrease in the renal contents of PGE2 compared to the positive group (Figure 5b). Notably, LMNS (400 mg/kg) showed a better effect than LME (400 mg/kg) in reducing the renal contents of PGE2.

#### 3.4.5. Effect on the MAPK/ERK Pathway

**Figure 6:**
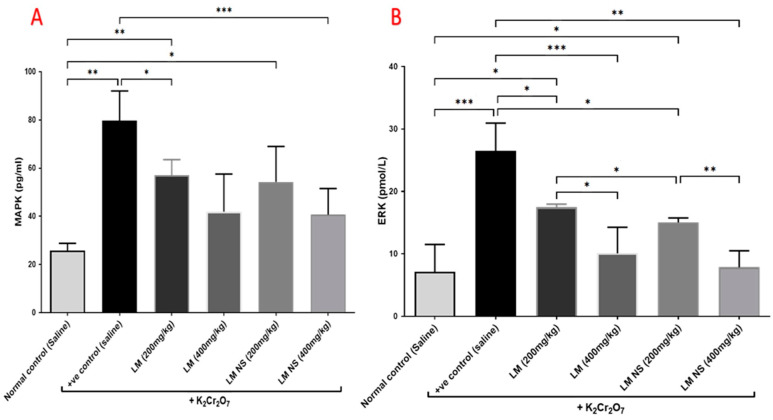
Effect of treatment with LME and LMNS on (**A**) MAPK and (**B**) ERK levels in kidney tissue under nephrotoxicity induced by a single intraperitoneal injection of K_2_Cr_2_O_7_ in rats. Data are expressed as (mean ± SD) where *n* = 6. Statistical analysis was performed using the one-way analysis of variance (ANOVA) followed by the Tukey’s multiple comparison test. * *p* ≤ 0.05, ** *p* ≤ 0.01, *** *p* ≤ 0.001, **** *p* ≤ 0.0001.

Administration of K_2_Cr_2_O_7_ led to significant increases in the MAPK and ERK levels in the kidney tissue compared with the negative control group. Meanwhile, the administration of LME ((200 and 400 mg/kg, respectively) or LMNS ((200 and 400 mg/kg, respectively) markedly (*p* < 0.05) decreased MAPK and ERK levels compared to the positive control group in a dose-dependent manner (Figure 6). No significant differences were observed between the LMNS and LME in reducing the renal MAPK and ERK levels.

#### 3.4.6. Histopathological Examination

##### Light Microscopic Observations

**Figure 7:**
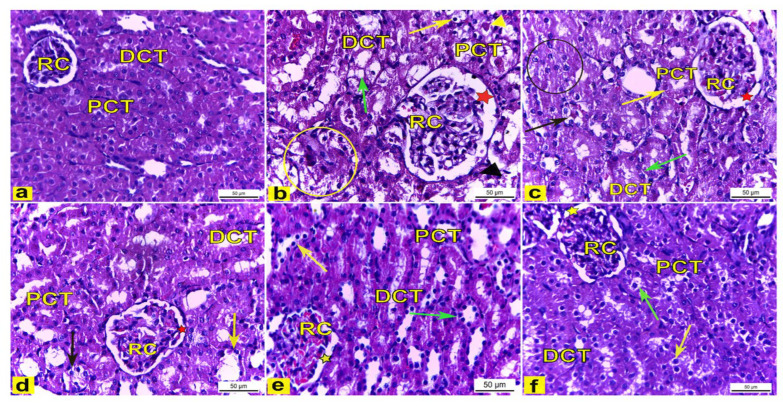
The renal cortex tissue of rats. H&E stain. X400. (**a**) The renal cortex of control rats showed normal renal corpuscles (RC), proximal convoluted tubules (PCT), and distal convoluted tubules (DCT). (**b**) Rats treated with K_2_Cr_2_O_7_ revealed an enlarged renal corpuscle (RC) with a wide renal space (red star), tubular degeneration (yellow circle), vacuolar degeneration (yellow arrow) of the proximal convoluted tubules (PCT) with a luminal cast (yellow arrowhead), and distal convoluted tubules (DCT) with a loss in the cytoplasmic content (green arrow) with the sloughing of the lining cuboidal cells (black arrowhead) into the tubular lumen. (**c**) Rats pretreated with 200 mg/kg LME had a less enlarged renal corpuscle (RC) with a narrower renal space (red star), with some PCTs (yellow arrow) and DCTs (green arrow) appearing with nearly normal histoarchitecture, but there was the tubular degeneration of several DCTs (black circle) and PCTs (black arrow) which displayed vacuolar degeneration. (**d**) Rats pretreated with 400 mg/kg LME exhibited a nearly normal-sized renal corpuscle (RC) with a normal renal space (red star), with most proximal (PCT) and distal (DCT) convoluted tubules appearing nearly normal except for a few DCTs (yellow arrow) and PCTs (black arrow) appearing degenerated. (**e**) Rats pretreated with 200 mg/kg LMNS and (**f**) 400 mg/kg LMNS showed a renal cortex had nearly normal renal corpuscles (RC) with a normal renal space (yellow star), and most of the proximal (PCT) and distal (DCT) convoluted tubules appeared to have nearly normal lining cells, except for a few proximal (green arrows) and distal (yellow arrows) convoluted tubules that displayed a loss in cytoplasmic acidophilia.

H&E-stained kidney tissues obtained from the control rats (Group I) showed a normal histoarchitecture of the renal cortex that consisted of a renal corpuscle with a normal diameter and housed glomerular capillaries, and enclosed with the Bowman’s capsule were the proximal convoluted tubules (PCT), which were lined with the truncated pyramidal cells with narrow lumina, and distal convoluted tubules (DCT) which were lined with the cuboidal cells with a wide lumina (Figure 7a). However, the renal tissue of K_2_Cr_2_O_7_-treated rats (Group II) displayed several histological changes of the renal cortex compared to the control rats. The renal cortex showed enlarged renal corpuscles with a wide renal space, tubular degeneration, while the proximal convoluted tubules displayed vacuolar degeneration with a luminal cast, and the distal convoluted tubules showed a complete loss of the cytoplasmic content with the sloughing of the lining cuboidal cells into the tubular lumen (Figure 7b). Meanwhile, rats pretreated with 200 mg/kg LME (Group III) displayed renal tissues with attenuated histopathological lesions compared to group II, as manifested by the less enlarged renal corpuscle with a narrower renal space, with several PCTs and DCTs appearing with a nearly normal histoarchitecture, though also accompanied with the tubular degeneration of a few distal convoluted tubules and a few proximal convoluted tubules showing vacuolar degeneration (as shown in Figure 7c). Moreover, rats pretreated with 400 mg/kg LME (Group IV) showed less structural alterations in the renal tissue compared with Group II. The renal cortex had a nearly normal-sized renal corpuscle with a normal renal space, and most proximal and distal convoluted tubules displayed a nearly normal cellular architecture except for a few tubules that appeared to be degenerated (Figure 7d). Furthermore, rats pretreated with 200 mg/kg and 400 mg/kg LMNS (Group V and VI), respectively, showed a marked recovery of the renal tissue compared to Group II. The renal cortex displayed nearly normal renal corpuscles accompanied with a normal renal space, and most of the proximal and distal convoluted tubules had nearly normal lining cells, except for a few proximal and distal convoluted tubules that had a loss of cytoplasmic acidophilia (Figure 7e,f).

##### Immunohistochemical Examination of Cyclo-Oxygenase 2 (COX-2)

**Figure 8:**
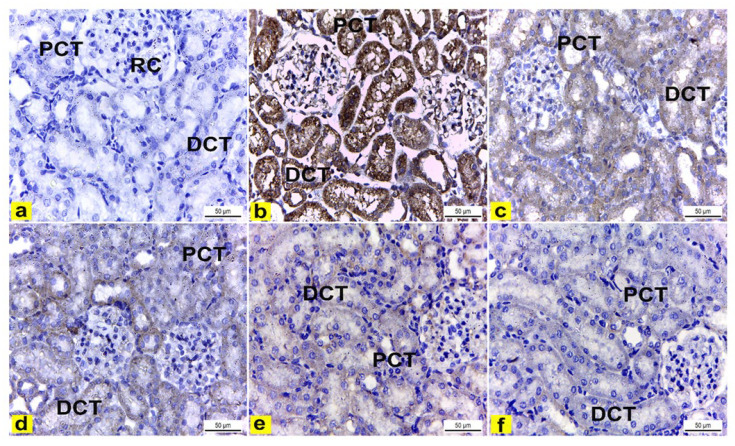
Immunohistochemically stained COX2 renal cortex sections of rats. X400. (**a**) Renal cortex of control rats showed negative COX2 immuno-expression in the cytoplasm of proximal (PCT) and distal (DCT) convoluted tubules. (**b**) Rats treated with K_2_Cr_2_O_7_ revealed strong positive COX2 immunoreaction in the cytoplasm of the proximal (PCT) and distal (DCT) convoluted tubules. (**c**) Rats pretreated with 200 mg/kg LME 200 displayed a moderate COX-2 immuno-expression in the cytoplasm of the proximal (PCT) and distal (DCT) convoluted tubules. (**d**) Rats pretreated with 400 mg/kg LME exhibited a mild level of COX-2 immuno-expression in the cytoplasm of the proximal (PCT) and distal (DCT) convoluted tubules. **e**) Rats pretreated with 200 mg/kg LMNS showed a mild level of COX-2 immunoreactivity in the cytoplasm of the proximal (PCT) and distal (DCT) convoluted tubules. (**f**) Rats pretreated with 400 mg/kg LMNS 400 revealed a negligible level of COX2 immunoreaction in the cytoplasm of the proximal (PCT) and distal (DCT) convoluted tubules.

**Figure 9:**
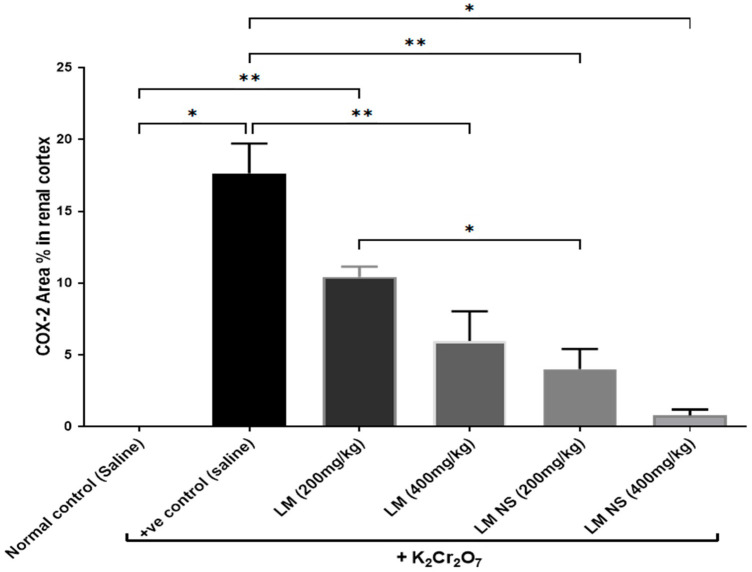
Effect of treatment with LME and LMNS on the percent area covered by COX2-positive immunoreactive cells within the renal cortex under nephrotoxicity induced by a single intraperitoneal injection of K_2_Cr_2_O_7_ in rats. Data are expressed as (mean ± SD) where *n* = 6. Statistical analysis was performed using the one-way analysis of variance (ANOVA), followed by the Tukey’s multiple comparison test. * *p* ≤ 0.05, ** *p* ≤ 0.01, *** *p* ≤ 0.001, **** *p* ≤ 0.0001.

The immunohistochemical examination of COX-2-stained renal tissue of control rats (Group I) showed negative immune-expression (Figure 8a). However, renal tissue of rats treated with K_2_Cr_2_O_7_ (Group II) showed a strong positive COX-2 immunoreaction in the cytoplasm of proximal and distal convoluted tubules that was significantly increased by 16.6 compared to control rats (Figure 8b and Figure 9). In contrast, rats pretreated with LME 200 mg/kg (Group III) showed a moderate COX-2 immuno-expression in the cytoplasm of proximal and distal convoluted tubules that significantly decreased by 10.4 compared to group II (Figure 8c and Figure 9). Meanwhile, rats pretreated with LME 400 mg/kg (Group IV) and LMNS 200 mg/kg (Group V) respectively displayed a mild COX-2 immunoreactivity in the cytoplasm of proximal and distal convoluted tubules that significantly reduced by 5.9 and 3.6 versus group II (Figure 8d,e and Figure 9) suggestive for the improved effect of LMSN compared to LME. Additionally, rats pretreated with LMNS 400 mg/kg (Group VI) had a negligible COX-2 immuno-expression in the cytoplasm of proximal and distal convoluted tubules that significantly diminished by 0.8 compared to group II (Figure 8f and Figure 9).

## 4. Discussion

Potassium dichromate (K_2_Cr_2_O_7_), is the most toxic form of Cr, and has been shown to induce nephrotoxicity. Post K_2_Cr_2_O_7_ administration, kidneys accumulate the highest concentration of hexavalent chromium, which is specifically deposited into the proximal convoluted tubules [40]. Immediately, K_2_Cr_2_O_7_ is reduced to trivalent chromium [Cr(III)], promoting reactive oxygen species (ROS) production via the Fenton reaction. Thus, oxidative stress has been predominantly linked to K_2_Cr_2_O_7_-induced nephrotoxicity [41]. In the current study, K_2_Cr_2_O_7_ administration at a single dose of 15 mg/kg BW caused renal dysfunction, as detected by the marked changes in the serum creatinine and urea. This result was deemed to be in agreement with El-Demerdash et al., 2021 [15]. Meanwhile, pretreatment with LME and LMNS alleviated the renal toxicity of K_2_Cr_2_O_7_, as manifested by the decreased levels in creatinine and urea.

According to Sharma et al., 2022 [42], chromium toxicity is associated with the production of ROS, which causes oxidative stress and disturbs the equilibrium between the oxidants and the antioxidants. Rats treated with K_2_Cr_2_O_7_ showed a notable reduction in the antioxidant enzyme catalase, which is crucial for preserving the cellular redox equilibrium, and preventing oxidative damage by converting hydrogen peroxide (H_2_O_2_) into water and oxygen, respectively. Catalase may be inhibited by the chromium attaching to the enzyme’s active site and/or by overusing the enzyme to neutralize the free radicals produced by the metal, which results in an irreversible inhibition of the enzyme’s activity [43]. In the present work, pretreatment with both LME and LMNS ameliorated the reduction in the catalase levels induced by K_2_Cr_2_O_7_. Therefore, LME exhibited a strong antioxidant scavenging activity, which was mostly attributed to its rich phenolic composition that was exemplified by flavonoids, such as luteolin, and phenolic acids, such as protocatechuic acid, both of which are potential antioxidants and thus protect the kidney from K_2_Cr_2_O_7_-induced oxidative damage and alleviate nephrotoxicity [44,45,46]. UDP-glucuronosyltransferase (UGT) is an essential enzyme in the metabolism and elimination of drugs and other xenobiotics from the body [47]. In the present study, K_2_Cr_2_O_7_ significantly downregulated UGT levels in the K_2_Cr_2_O_7_-treated group compared with the control group, which was likely attributed to either the disruption of the structure, or by altering the catalytic activity of UGT enzymes by K_2_Cr_2_O_7,_ leading to interference with their normal functioning [48]. Pretreatment with both LME and LMNS prevented the reduction in the UGT levels relative to the K_2_Cr_2_O_7_-treated group. LME is rich in antioxidant phytochemicals, including especially the flavonoids and phenolic acids, which promote the induction of phase II enzymes, such as UGT through a variety of signaling pathways, thereby enhancing metabolism, xenobiotic detoxification, and antioxidant and free radical scavenging activity capacity [49]. Numerous ligand-activated transcription factors, particularly those belonging to the nuclear receptor superfamily and their natural or synthetic ligands, have been demonstrated to activate these UGT genes. Nuclear factor erythroid-related factor 2 (Nrf2) is one of these factors [50,51]. Nrf2, a key redox regulator, controls antioxidant genes and phase II detoxifying enzymes to maintain the intracellular redox equilibrium, and to further exert a significant protective effect against oxidative stress [52]. Rats treated with K_2_Cr_2_O_7_ showed a marked reduction in Nrf2 levels compared to the control rats. A previous study reported that K_2_Cr_2_O_7_ decreased the expression of Nrf2 in lung tissue, which was likely mediated via the inhibition of the Akt/GSK-3β/Fyn signaling pathway [53]. Both LME and LMNS mitigated against reductions in the Nrf2 level compared to the K_2_Cr_2_O_7_-treated group, which was indicated to be one of the involved action mechanisms. This protective effect may be due to the rich flavonoid content in LME, such as apigenin and luteolin, both of which have proven to increase the Nrf2 level in human hepatoma HepG2 cells [54].

Another explanation of renal injury induced by dichromate is through the modulation of the inflammatory process, as manifested by the increased COX-2 and PGE2 levels in the K_2_Cr_2_O_7_ group relative to the control rats. Studies have shown that K_2_Cr_2_O_7_ enhances the thyroid expression of COX-2, an inducible enzyme that catalyzes the conversion of arachidonic acid to prostaglandins, which are involved in inflammation and pain progression [55]. ROS generated by K_2_Cr_2_O_7_ have the power to significantly alter the regulation of redox-sensitive genes, including the upregulation of COX-2 [55,56]. In this study, pretreatment with LME and LMNS limited the elevation in COX-2 and PGE2 levels and reduced the inflammation relative to the K_2_Cr_2_O_7_-treated group. The anti-inflammatory effect of LME may be likewise attributed to flavonoids and phenolic acids, such as luteolin, apigenin, and protocatechuic acid which were all found to abrogate the inflammatory response through the inhibition of COX-2 and PGE2 [57,58].

The mitogen-activated protein kinase (MAPK) pathway is triggered when intracellular ROS levels are consistently elevated [59]. This explains why the K_2_Cr_2_O_7_-treated group in this study displayed the higher MAPK and ERK levels. According to Yin et al., 2019 [60], K_2_Cr_2_O_7_-induced kidney damage is mediated by the overexpression of MAPK and ERK. Hexavalent chromium has also been reported to promote ROS production and activate the MAPK/ERK pathway, which was shown to mediate the overexpression of COX2 and the production of PGE2 [61,62]. As opposed to the group treated with K_2_Cr_2_O_7_, pretreatment with LME and LMNS reduced the MAPK and ERK levels in the renal tissue. Protocatechuic acid, a phenolic acid in LME, was found to reduce kidney damage induced by lipopolysaccharides through the downregulation of the MAPK/ERK signaling pathway [63]. Furthermore, apigenin and luteolin, the two major flavonoids detected in LME, inhibited the MAPK/ERK pathway in LPS-stimulated microglia cells [64]. LMNS demonstrated a better activity than LME in reducing the levels of urea, creatinine, COX-2, and PGE2, as well as in restoring UGT levels. This could be due to the enhancement of the biological effects of LME by silver nanoparticles through the augmentation of the active biomolecule’s concentration [16].

In the present study, the renal tissue of K_2_Cr_2_O_7_-treated rats (Group II) displayed several histological changes in the renal cortex compared with the control rats. The renal cortex had enlarged renal corpuscles with a wide renal space, tubular degeneration, and proximal convoluted tubules which displayed vacuolar degeneration with a luminal cast, and distal convoluted tubules which showed the complete loss in the cytoplasmic content with the sloughing of the lining cuboidal cells into the tubular lumen. These findings are in accordance with previous reports [65,66,67], which were suggestive that K_2_Cr_2_O_7_-induced toxicity and the induction of oxidative stress and apoptosis arise through the generation of reactive oxygen species (ROS). Consequently, ROS triggered K_2_Cr_2_O_7_ toxicity owing to the reduction of hexavalent chromium to the trivalent form, thereby inducing damage to the cellular components [68]. Importantly, the unique molecular structure of the identified chlorogenic acid with its multiple active hydroxyl groups enhances its antioxidant capacity. Moreover, it effectively attenuated the activation of the NF-κB signaling pathway either directly or indirectly leading to the blockage in the expression of several pro-inflammatory factors, such as interleukin 6 (IL-6), TNF-α, and interleukin 1β (IL-1β) at the gene level [69]. Similarly, Cheng et al. [70] highlighted the potential activity of chlorogenic acid through the inhibition of the Pb-induced increase of cytoplasmic NF-κB, Bax, cytochrome C, and caspase-9 protein expressions.

Of note, Veeren et al. [71] reported the nephroprotective potential of caffeic acid, the major phenolic acid of *Antirhea borbonica* mediated through the downregulation of the pro-inflammatory molecules associated with the elevation in Nrf2 mRNA expression and CAT enzyme activity, which was likewise detected using UPLC-MS in LME.

Recently, luteolin has shown a good reno-protective against renal injury caused by multiple stimuli, such as renal ischemia, and nephrotoxic drugs mediated via its antioxidant potential [72]. Importantly, apigenin and its glycosides, which have been identified as major flavones in the *L. mucronata* extract are considered as one of the most potential flavonoids containing a myriad of effects, such as anti-inflammatory, antioxidant, anti-cancer, and anti-hypertensive effects. Recently, Alam et al. [73] highlighted the promising protective effect of apigenin against cisplatin-induced nephrotoxicity. Moreover, it effectively protected the renal mitochondria against carbon nanotube-induced mitochondrial dysfunction [73].

With regard to coumarins, another phenolic subclass detected in LME, and more specifically esculetin, the unique *Launaea* coumarin, has been reported to exert a higher antioxidant and anti-inflammatory potential than other coumarins. It was able to prevent apoptosis in tert-butyl hydroperoxide-induced oxidative stress in HEK294 cells which is involved in chronic kidney diseases [74]. The production of advanced glycation end products (AGEs), the disruption of proinflammatory cytokines, and the activation of cellular pathways, such as the TGF-β/Smad, NF-kB (p65), and NLRP3/Caspase 1 axis, have all been reported to be decreased by coumarins [75,76]. According to the mounting evidence, coumarins may also prevent the kidneys from producing extracellular matrix components, and from activating myofibroblasts that secrete α-smooth muscle action [77].

Unfortunately, most of the bioactive metabolites, including phenolic acids, flavonoids, and coumarins are lipophobic, and their use has been limited due to their low bioavailability, poor absorption, and tailored distribution to the target site. Herbal nano-formulations can provide avenues to improve their cellular uptake and increase their transport from the blood stream to the kidneys [78]. Interestingly, our findings were in line with [79], who highlighted the promising antioxidant activity of the encapsulated apigenin for the treatment of hepatocellular carcinoma with an excellent stability and bioavailability. In the same context, the authors of [80] confirmed that the crystal structure of quercetin nanoparticles demonstrated a higher bioavailability than quercetin itself due to the increased surface area with a higher solubility. Notably, quercetin nanoparticles were able to inhibit the expression of profibrogenic genes.

In our study, LMNS at a dose of 400 mg/kg exhibited a higher activity in the reduction of renal PGE2, and serum urea and creatinine compared to a similar dose of LME. Regarding the histopathological examination, LMNS treatment at both doses demonstrated a marked recovery of the renal tissue with the renal cortex having nearly normal corpuscles with a normal renal space. Future studies should now focus on identifying active agents in these nano-formulations using isolation and spectroscopic techniques in order for the results to be conclusive.

Histopathological and immunohistochemical assays as another measure type for the demonstration of nephrotoxic and protective effects revealed enlarged renal corpuscles that were observed in this study, coming in line with Saber et al. and Stoev et al., [81,82] who hypothesized that an increased glomerular size may be attributed to glomerular endothelial proliferation. In contrast, pretreatment with LME and LMNS demonstrated the amelioration of K_2_Cr_2_O_7_ nephrotoxicity, which was evident in the form of decreased histological lesions in the rat renal tissue. This amelioration was clear and marked in rats the pretreated with LME and LMNS, as the renal cortex had nearly normal renal corpuscles, and most of the proximal and distal convoluted tubules had nearly normal lining cells, thereby indicating the powerful antioxidant effect of LME.

Likewise, proximal convoluted tubules exhibited vacuolar degeneration with a luminal cast that was consistent with Hegazy et al. [83], who reported the tubular damage and nephrotoxic effects of chromate owing to its accumulation in vacuoles inside the proximal tubular cells, causing the slow excretion and staying of Cr in the kidney.

Furthermore, immunohistochemically, the renal tissue of rats treated with K_2_Cr_2_O_7_ (Group II) showed strong positive COX-2 immunoreactions in the cytoplasm of the proximal and distal convoluted tubules compared to the control rats. These results are in accordance with Salama et al. [84], who reported an increase in COX-2 immuno-expression in the brain and lung tissues of rats that received a single i.p. injection of K_2_Cr_2_O_7_ (15 mg/kg). Our results indicated that K_2_Cr_2_O_7_ likewise exhibits an inflammatory effect on the renal tissue. Meanwhile, rats pretreated with LME and LMNS displayed a significant decrease in COX2 immunoreaction in the renal tissue, with highest effects observed in the case of rats pretreated with 200mg/kg LMNS.

## 5. Conclusions

**Figure 10:**
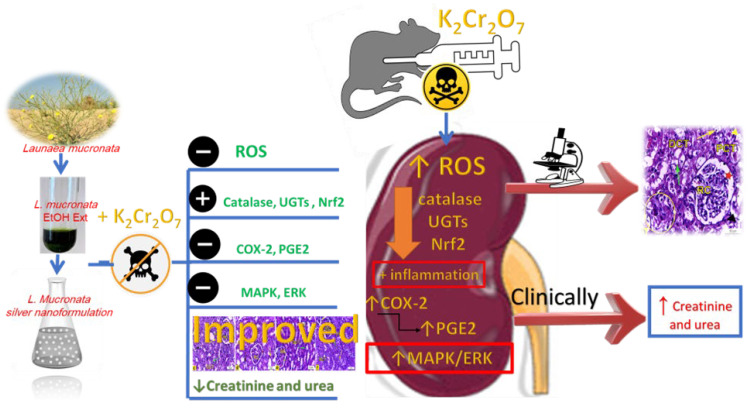
Summarized diagram of the LME and LMNS’ nephrotoxicity action pathway against K_2_Cr_2_O_7_-intoxicated rats.

The present investigation has been regarded as the first examination of the possibility of LME and LMNS against K_2_Cr_2_O_7_-intoxicated rats. The LME and LMNS dosing prior to K_2_Cr_2_O_7_ significantly improved the renal tissue architecture, while also restoring the levels of most biochemical indicators through inhibiting the MAPK/ERK pathway, activating Nrf2, and altering antioxidant and metabolic enzymes (Figure 10). These findings imply that LME and LMNS both provide nephroprotection against K_2_Cr_2_O_7_-induced toxicity, which could be attributed to the chemical components of flavonoids, phenolic acids, organic acids, coumarins, and fatty acids characterized using UPLC-ESI-qTOF-MS. Therefore, the current findings support the conclusion that LME and LMNS could be prospective agents and an innovative form of defense against nephrotoxicity. To demonstrate the effects of the active compounds identified in the extract, subsequent research should standardize and evaluate individual components.

## Figures and Tables

**Table 1:** Tentative identification of LME metabolites by UPLC-qTOF-MS in their negative and positive ion modes.

No.	Rt.	*m*/*z*	[M−H]^−^/[M+H]^+^	MS/MS	Error (ppm)	Tentative Identification
1	0.951	191.0561	C_7_H_11_O_6_^−^	127	−2.54	Quinic acid
2	1.003	133.0142	C_4_H_5_O_5_^−^	115	4.08	Malic acid
3	1.059	290.0881	C_11_H_16_NO_8_^−^	200, 128	−8.11	Deoxy-dehydro-*N*-acetyl neuraminic acid
4	1.493	167.0197	C_4_H_7_O_7_^−^	124	−8.18	Tartaric acid
5	2.569	163.0401	C_9_H_7_O_3_^−^	145, 119	−1.42	*P*-coumaric acid
6	2.843	169.0142	C_7_H_5_O_5_^−^	125	−2.05	Gallic acid
7	3.558	341.0878	C_15_H_17_O_9_^−^	179, 161	−0.57	Caffeoyl hexose
8	7.426	315.0722	C_13_H_15_O_9_^−^	153	−4.89	Protocatechuic acid-*O*-hexoside
9	7.565	137.0244	C_7_H_5_O_3_^−^	119	−2.05	*p*-hydroxy benzoic acid
10	8.137	311.0409	C_13_H_11_O_9_^−^	179, 149	−0.78	Caffeoyl tartaric acid
11	8.402	137.0244	C_7_H_6_O_3_^−^	93	−0.6	Salicylic acid
12	8.537	353.0878	C_16_H_17_O_9_^−^	191	−1.68	Chlorogenic acid
13	8.610	325.0929	C_15_H_17_O_8_^−^	191, 173	−1.56	Coumaroyl hexoside
14	8.680	177.019	C_9_H_5_O_4_^−^	89	−1.5	Aesculetin
15	8.710	339.0722	C_15_H_15_O_9_^−^	177	−0.72	Aesculin
16	8.854	313.0929	C_14_H_17_O_8_^−^	179	0.35	Unknown caffeic acid derivative
17	9.353	179.035	C_9_H_7_O_4_^−^	135	−1.76	Caffeic acid
18	9.722	515.0831	C_24_H_19_O_13_^−^	353	−0.36	Chlorogenic acid hexoside
19	9.827	611.1607	C_27_H_31_O_16_^+^	449, 287	2.56	Luteolin- dihexoside
20	10.134	595.1657	C_27_H_31_O_15_^+^	449, 287	3.44	Luteolin-*O*-rutinoside
21	10.152	461.0725	C_21_H_17_O_12_^−^	285	0.11	Luteolin-*O*-glucuronic acid
22	10.277	447.0933	C_21_H_19_O_11_^−^	285	1.87	Luteolin-*O*-hexoside
23	10.291	443.1042	C_15_H_23_O_15_^−^	285	4.15	Unknown luteolin derivative
24	10.349	195.0652	C_10_H_11_O_4_^+^	179	0.95	Ferulic acid
25	10.451	515.1195	C_25_H_23_O_12_^−^	353, 191	0.97	Di-caffeoylquinic acid
26	10.464	197.1172	C_11_H_16_O_3_^+^	179, 133	1.64	Unknown phenolic acid
27	10.745	433.1129	C_21_H_21_O_10_^+^	271	2.37	Apigenin-*O*-hexoside
28	10.670	445.0776	C_21_H_17_O_11_^−^	269	−1.04	Unknown apigenin derivative
29	10.758	447.0922	C_21_H_19_O_11_^+^	271	2.44	Apigenin-*O*-glucuronic acid
30	10.934	187.0976	C_9_H_15_O_4_^−^	143	−0.09	Nonanedioic acid
31	11.004	211.0965	C_11_H_15_O_4_^+^	179	8.02	Unknown caffeic acid derivative
32	11.129	227.1278	C_12_H_19_O_4_^+^	209	1.26	Hydroxy jasmonic acid
33	11.618	461.1078	C_22_H_21_O_11_^+^	271	1.6	Unknown apigenin derivative
34	11.823	285.0405	C_15_H_9_O_6_^−^	257, 199	−2.58	Luteolin
35	11.889	571.0882	C_30_H_19_O_12_^−^	285	−1.57	Unknown Luteolin derivative
36	12.182	209.1536	C_13_H_21_O_2_^+^	173	1.47	Tridecatrienoic acid
37	12.515	269.0455	C_15_H_9_O_5_^−^	117	−2.42	Apigenin
38	12.540	331.2479	C_18_H_35_O_5_^+^	313	2.42	Trihydroxy-octadecenoic acid
39	12.549	337.0354	C_18_H_9_O_7_^−^	269	5.85	Unknown apigenin derivative
40	13.087	301.202	C_16_H_29_O_5_^−^	283	0.49	Hydroxy hexadecanedioic acid
41	13.22	287.2228	C_16_H_31_O_4_^−^	269	−1.79	Dihydroxy hexadecanoic acid
42	13.782	941.5162	C_45_H_83_O_16_P_2_^−^	-	7.3	Phosphatidylinositol phosphate (18:0/18:2)
43	14.648	318.3003	C_18_H_40_NO_3_^+^	303	1.8	Amino octadecanetriol
44	17.273	295.2268	C_18_H_31_O_3_^−^	277	1.6	Hydroxy octadecdienoic acid
45	19.08	271.2279	C_16_H_31_O_3_^−^	225	−1.22	Hydroxy-palmitic acid

## Data Availability

Not Applicable.

## Supplementary Materials

The following supporting information can be downloaded at: https://www.mdpi.com/article/10.3390/metabo13070786/s1, Figure S1: Experimental design and time cycle; Figure S2: Negative mode MS/MS spectrum of chlorogenic acid hexoside; Figure S3: Negative mode MS/MS spectrum of caffeoyl hexoside; Figure S4: Negative mode MS/MS spectrum of protocatechuic acid hexoside; Figure S5: Negative mode MS/MS spectrum of aesculin; Figure S6: Negative mode MS/MS spectrum of aesculetin; Figure S7: Negative mode MS/MS spectrum of luteolin-*O*-glucuronic acid; Figure S8: Negative mode MS/MS spectrum of luteolin-*O*-hexoside; Figure S9: Negative mode MS/MS spectrum of luteolin; Figure S10: Negative mode MS/MS spectrum of apigenin-*O*-hexoside; Figure S11: Negative mode MS/MS spectrum of quinic acid; Figure S12: Negative mode MS/MS spectrum of salicylic acid; Figure S13: Negative mode MS/MS spectrum of tartaric acid; Figure S14: Negative mode MS/MS spectrum of coumaroyl hexoside; Figure S15: Negative mode MS/MS spectrum of malic acid; Figure S16: Negative mode MS/MS spectrum of *P*-hydroxy benzoic acid; Figure S17: Negative mode MS/MS spectrum of *P*-coumaric acid; Figure S18: Negative mode MS/MS spectrum of gallic acid; Figure S19: Negative mode MS/MS spectrum of chlorogenic acid; Figure S20: Negative mode MS/MS spectrum of dicaffeoylquinic acid; Figure S21: Negative mode MS/MS spectrum of ferulic acid; Figure S22: Negative mode MS/MS spectrum of caffeic acid; Figure S23: Negative mode MS/MS spectrum of caffeic acid derivative; Figure S24: Negative mode MS/MS spectrum of caffeoyl tartaric acid; Figure S25: Negative mode MS/MS spectrum of unknown phenolic acid; Figure S26: Negative mode MS/MS spectrum of hydroxy jasmonic acid; Figure S27: Negative mode MS/MS spectrum of deoxydehydro-*N*-acetylneuraminic acid; Figure S28: Negative mode MS/MS spectrum of nonanedioic acid; Figure S29: Negative mode MS/MS spectrum of apigenin; Figure S30: Positive mode MS/MS spectrum of luteolin-*O*-dihexoside; Figure S31: Positive mode MS/MS spectrum of luteolin-*O*-rutinoside; Figure S32: Negative mode MS/MS spectrum of unknown luteolin derivative; Figure S33: Negative mode MS/MS spectrum of unknown luteolin derivative; Figure S34: Negative mode MS/MS spectrum of unknown apigenin derivative; Figure S35: Positive mode MS/MS spectrum of unknown apigenin derivative; Figure S36: Negative mode MS/MS spectrum of unknown apigenin derivative; Figure S37: Negative mode MS/MS spectrum of trihydroxy octadecanoic acid; Figure S38: Negative mode MS/MS spectrum of hydroxy palmitic acid; Figure S39: Positive mode MS/MS spectrum of hydroxy linoleic acid; Figure S40: Negative mode MS/MS spectrum of hydroxy hexadecanedioic; Figure S41: Negative mode MS/MS spectrum of tridecatrienoic acid; Figure S42: Negative mode MS/MS spectrum of dihydroxy hexadecanoic; Figure S43: Positive mode MS/MS spectrum of amino octadecanetriol; Figure S44: Negative mode MS/MS spectrum of phosphatidylinositol phosphate.
