# Supplementary material for: Protective Action Mechanisms of Launaea mucronata Extract and Its Nano-Formulation against Nephrotoxicity in Rats as Revealed via Biochemical, Histopathological, and UPLC-QTOF–MS/MS Analyses
Source: Metabolites. 2023 Jun 23;13(7):786. doi: 10.3390/metabo13070786 (PMC10384424; doi:10.3390/metabo13070786)
Supplement: Supplementary file 1 [file metabolites-13-00786-s001.zip › metabolites-2448449-supplementary.pdf]

Supporting data

Protective Action Mechanisms of *Launaea Mucronata* Extract and Its Nano-Formulation against Nephrotoxicity in Rats as Revealed *via* Biochemical, Histopathological and UPLC-QTOF–MS/MS Analyses

Amany A. El-Fadaly [1,†], Inas Y. Younis [2,†], Mohamed F. Abdelhameed [1,†], Yasmine H. Ahmed [3], Tamer I.M. Ragab [4], Abd El-Nasser G. El Gendy [5], Mohamed A. Farag [2], Abdelsamed I. Elshamy [6,*] and Abdelbaset M. Elgamal [4,*]

1 Pharmacology Department, National Research Centre, 33 El Bohouth St., Dokki, Giza 12622, Egypt; aa.elfadaly@nrc.sci.eg (A.A.E-F.), mf.abdelhameed@nrc.sci.eg (M.F.A.)
2 Pharmacognosy Department, Faculty of Pharmacy, Cairo University, Kasr el Aini St., Cairo 11562, Egypt; inas.younis@pharma.cu.edu.eg (I.Y.Y.), mohamed.farag@pharma.cu.edu.eg (M.A.F.)
3 Department of Cytology and Histology, Faculty of Veterinary Medicine, Cairo University, Giza, 12211, Egypt; yasmine_hamdi@cu.edu.eg (Y.H.A.)
4 Department of Chemistry of Microbial and Natural Products, National Research Centre, 33 El-Bohouth St., Dokki, Giza 12622, Egypt; ti.ragab@nrc.sci-eg (T.I.M.R.)
5 Medicinal and Aromatic Plants Research Department, National Research Centre, Dokki, Giza 12622, Egypt; ag.el-gendy@nrc.sci.eg (A-E. G. E-G.)
6 Chemistry of Natural Compounds Department, National Research Centre, 33 El Bohouth St., Dokki, Giza 12622, Egypt
* Correspondence: algamalgene@yahoo.com (A.M.E.); elshamynrc@yahoo.com (A.I.E.); Tel.: +20-100-155-8689 (A.M.E.); +20-100-552-5108 (A.I.E.)
† These authors contributed equally to this work.

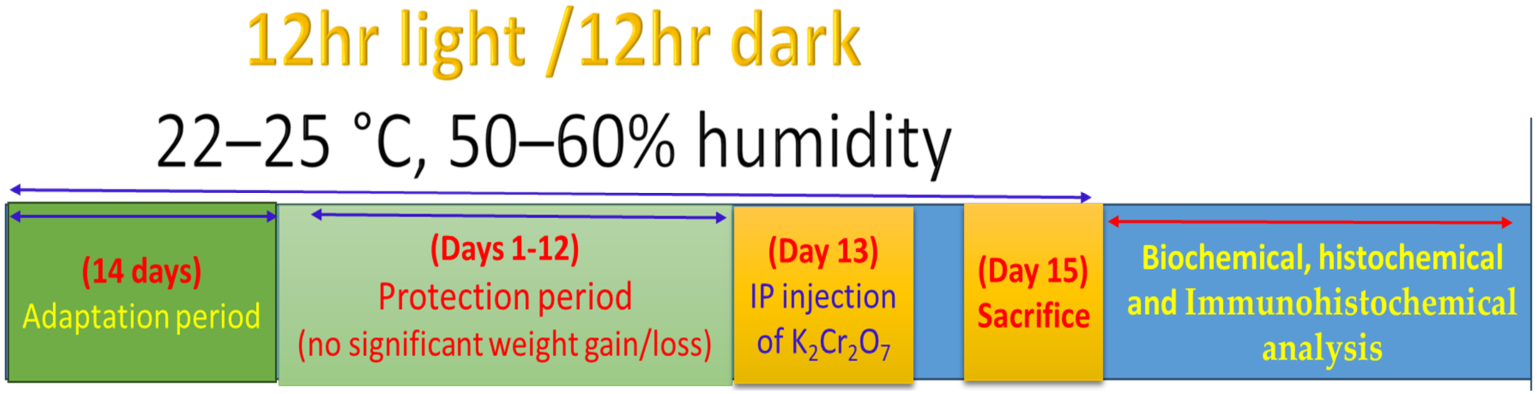

Figure S1. Experimental design and time cycle.

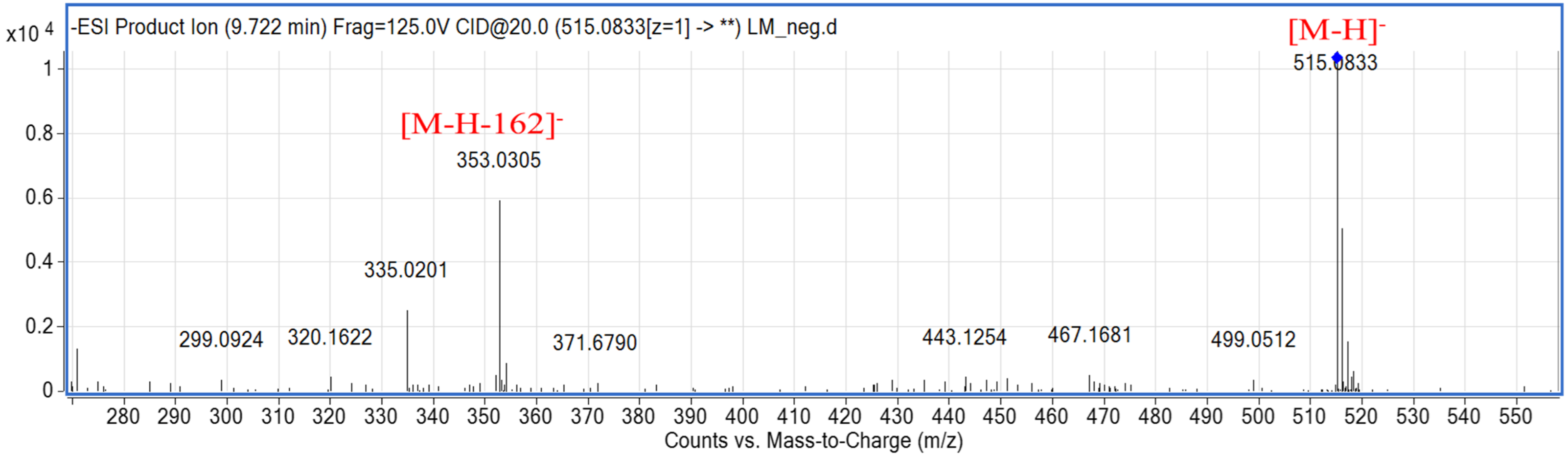

Figure S2. Negative mode MS/MS spectrum of chlorogenic acid hexoside.

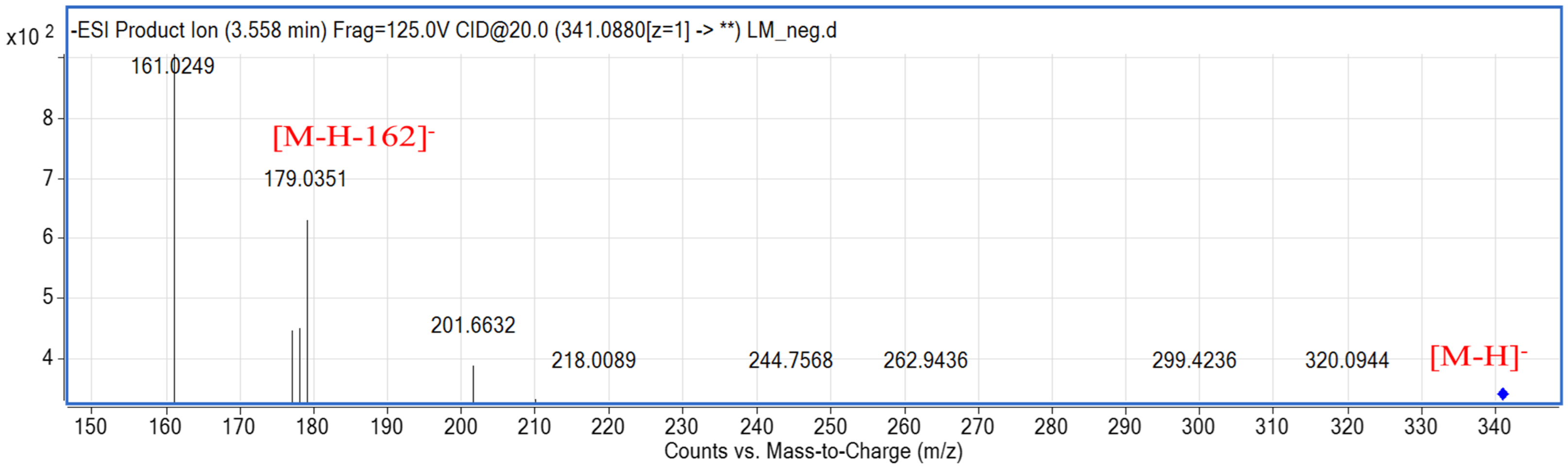

Figure S3: Negative mode MS/MS spectrum of caffeoyl hexoside.

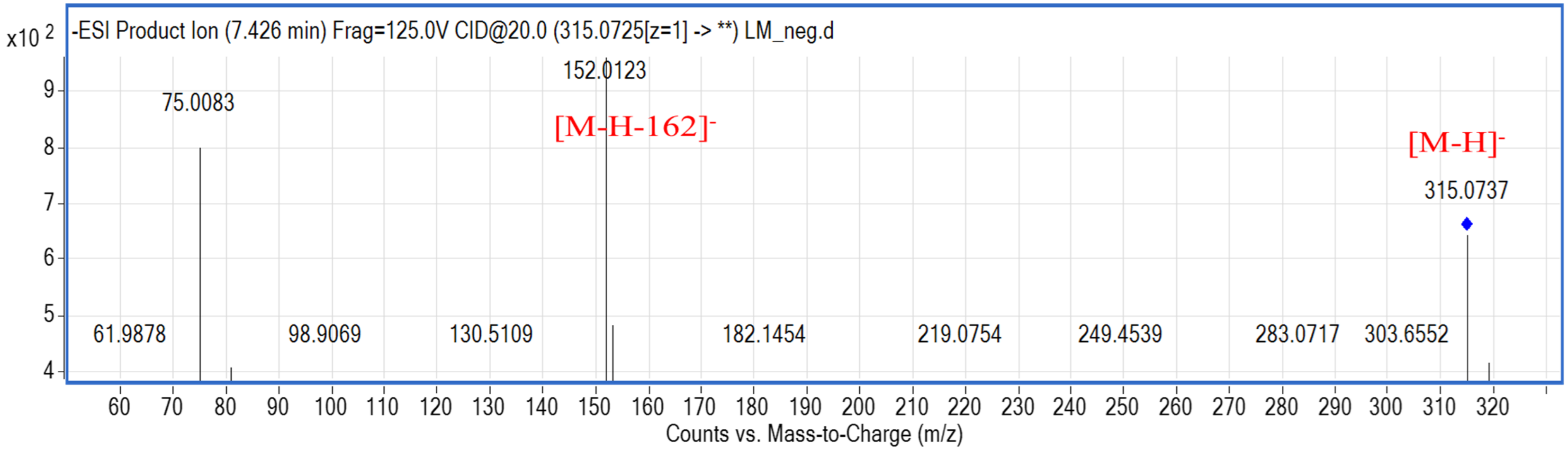

Figure S4: Negative mode MS/MS spectrum of protocatechuic acid hexoside.

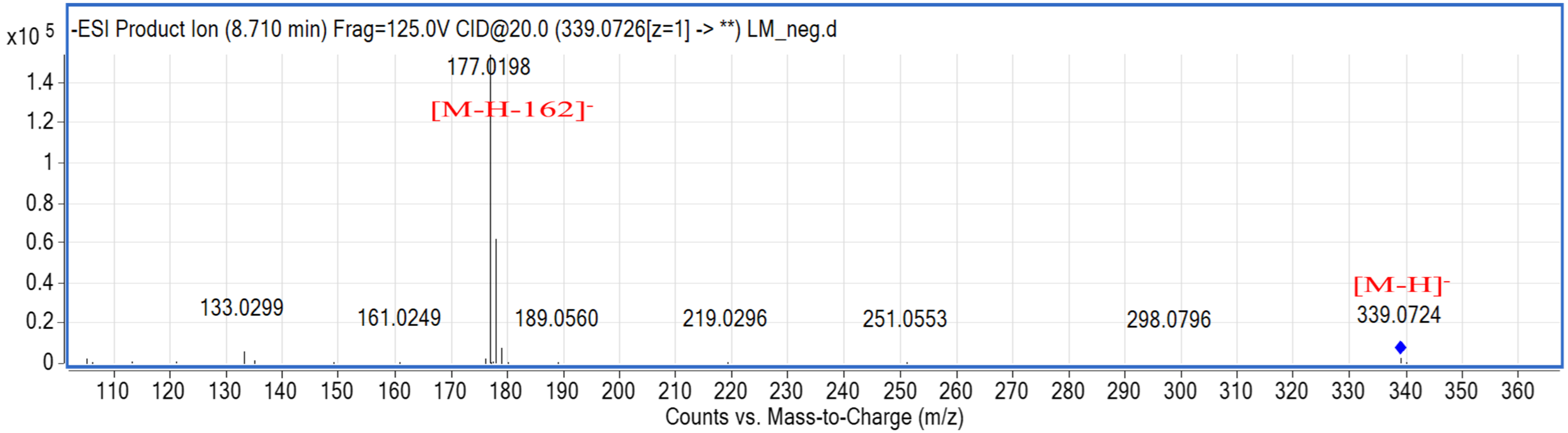

Figure S5: Negative mode MS/MS spectrum of aesculin.

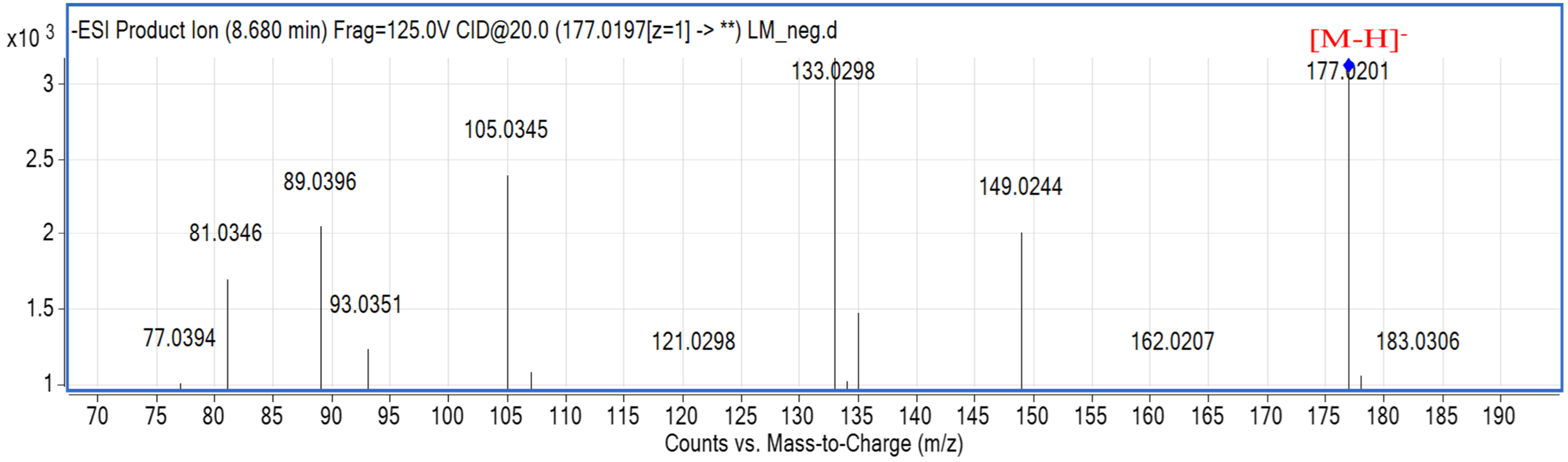

Figure S6: Negative mode MS/MS spectrum of aesculetin.

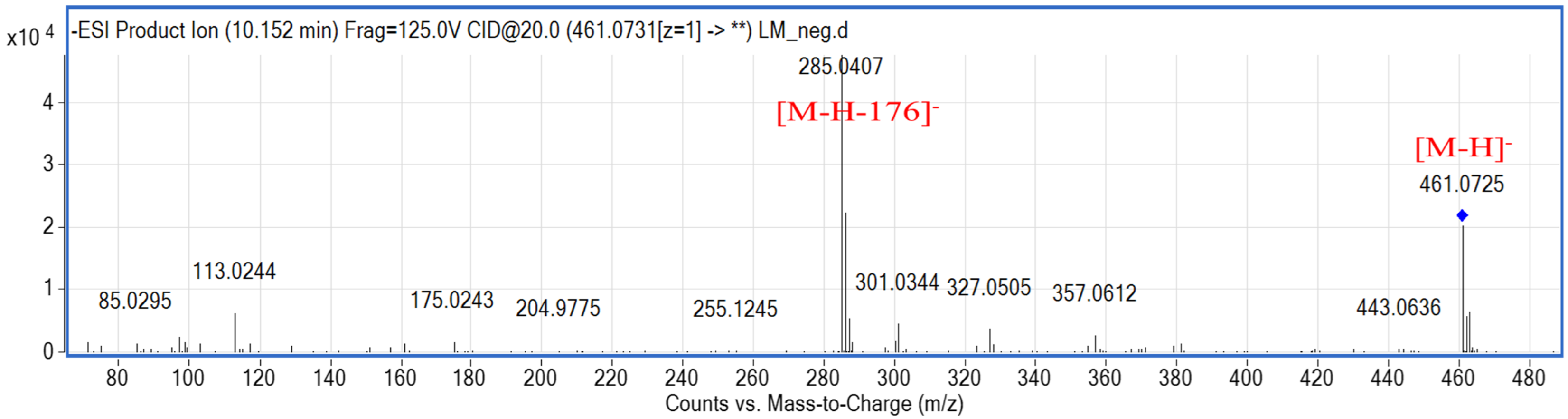

Figure S7: Negative mode MS/MS spectrum of luteolin-*O*-glucuronic acid.

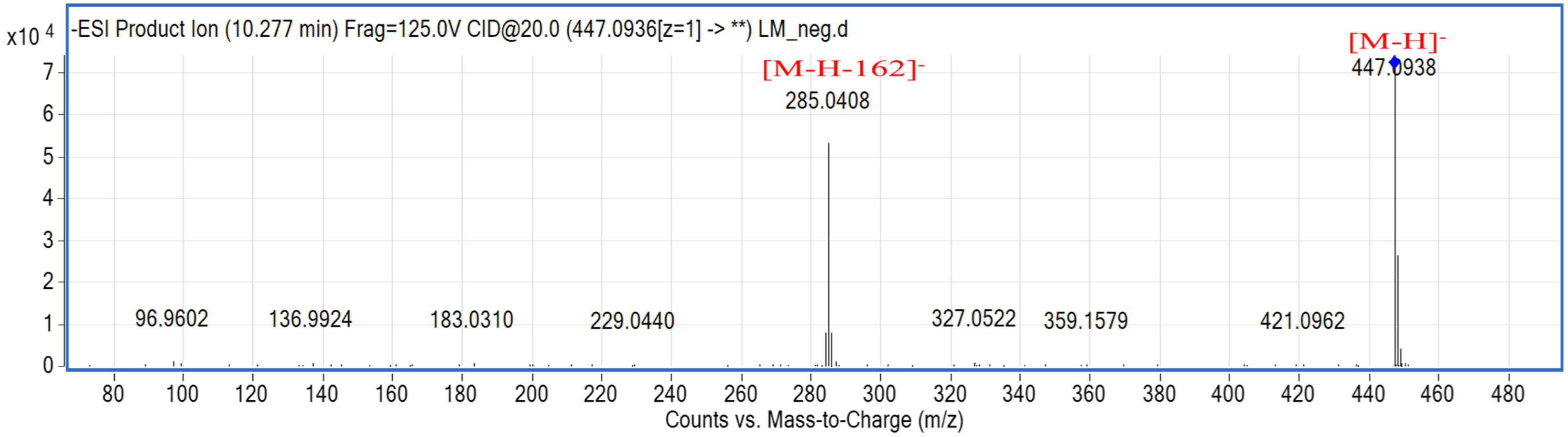

Figure S8: Negative mode MS/MS spectrum of luteolin-*O*-hexoside.

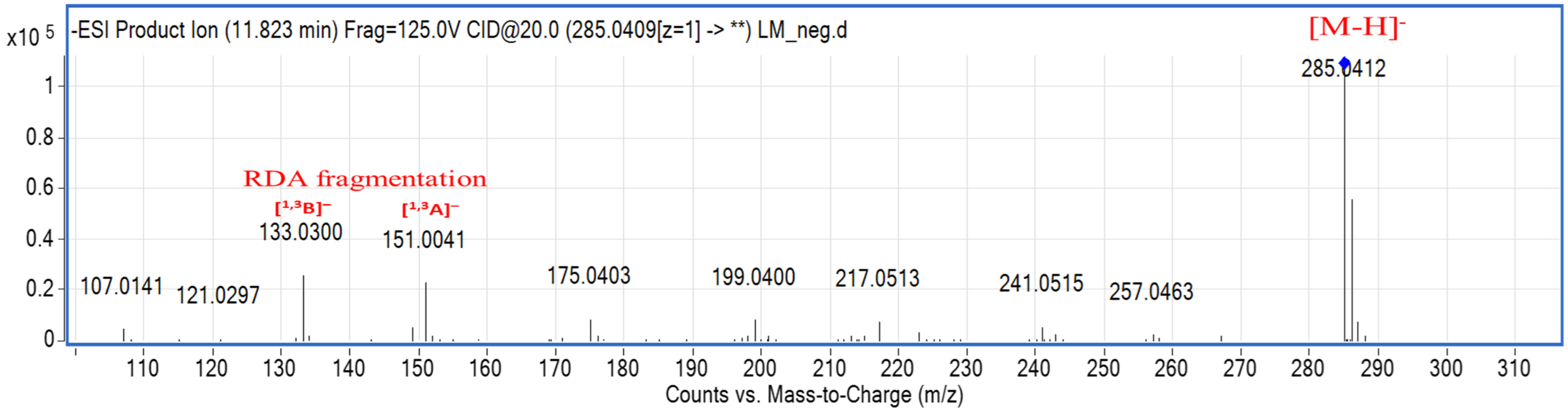

Figure S9: Negative mode MS/MS spectrum of luteolin.

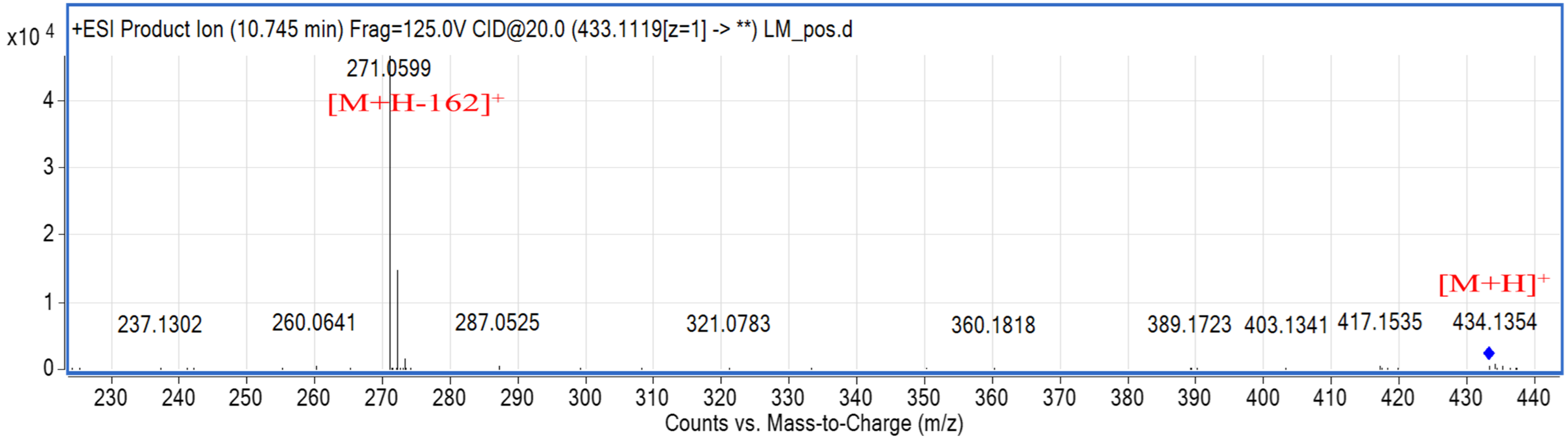

Figure S10: Negative mode MS/MS spectrum of apigenin-*O*-hexoside.

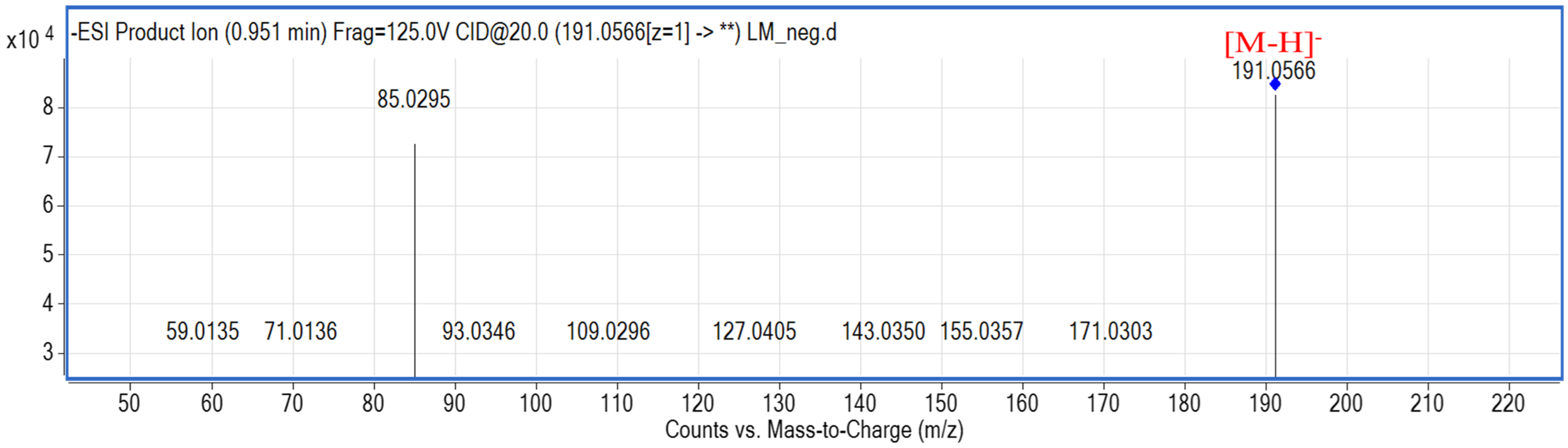

Figure S11: Negative mode MS/MS spectrum of quinic acid.

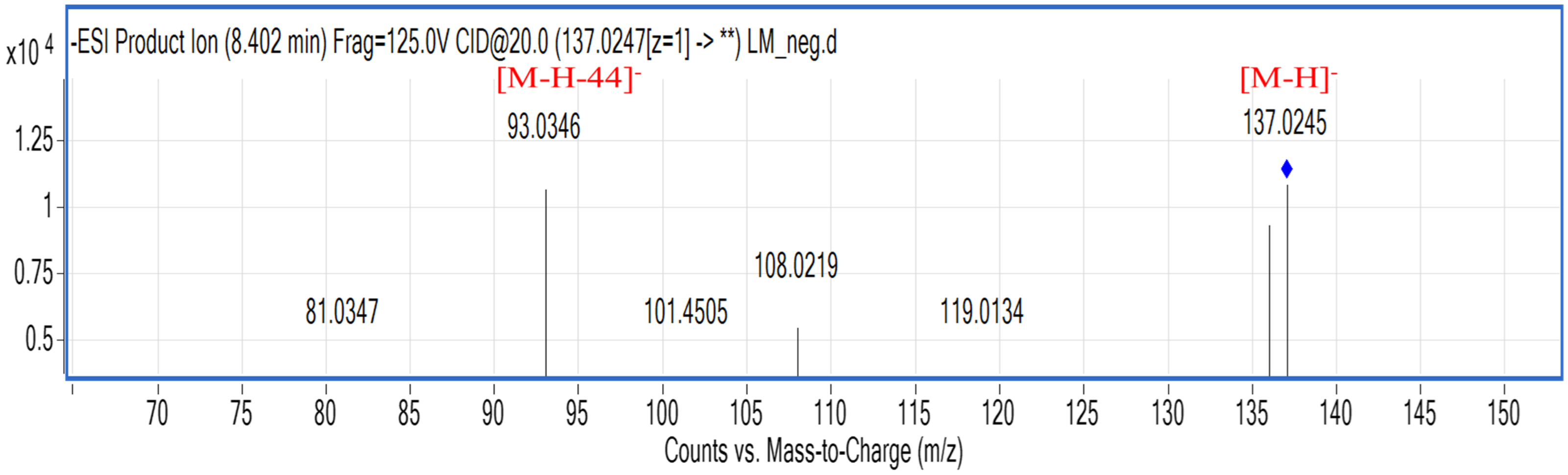

Figure S12: Negative mode MS/MS spectrum of salicylic acid.

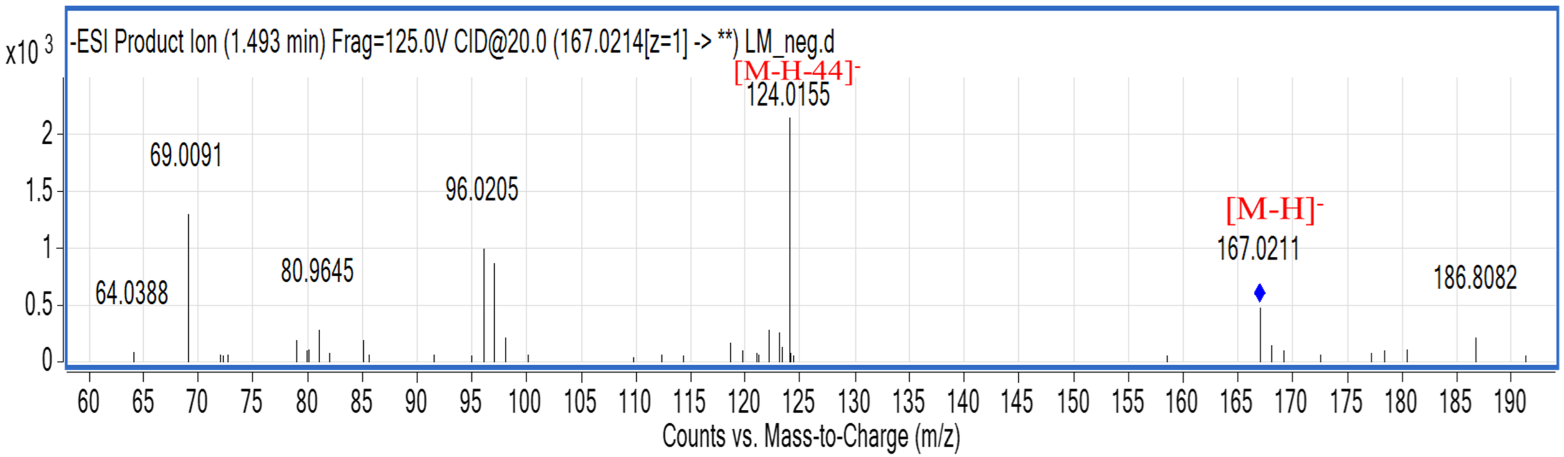

Figure S13: Negative mode MS/MS spectrum of tartaric acid.

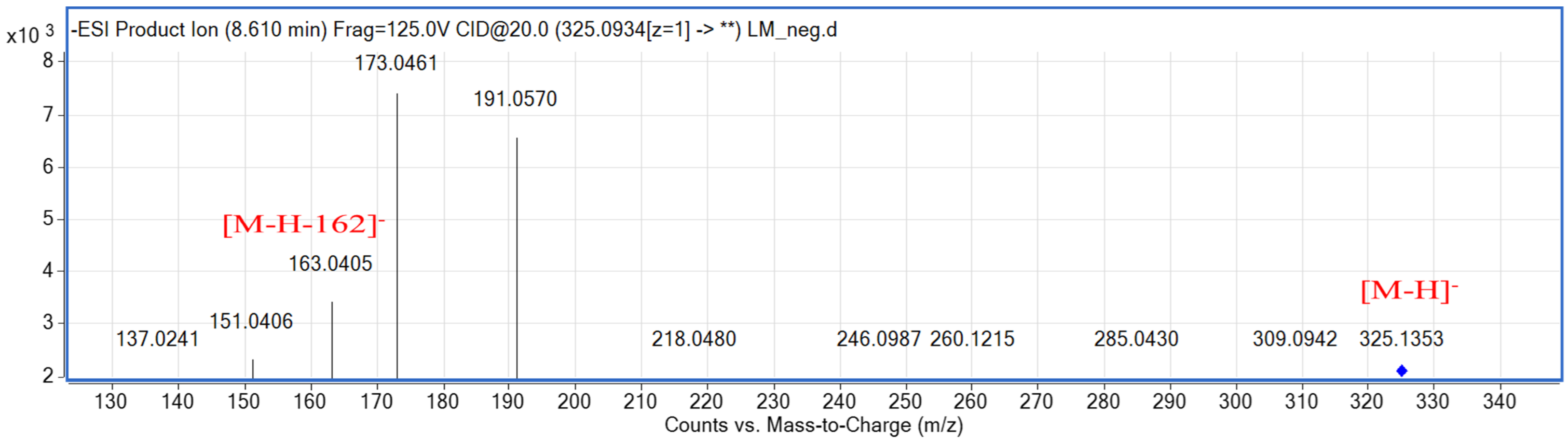

Figure S14: Negative mode MS/MS spectrum of coumaroyl hexoside.

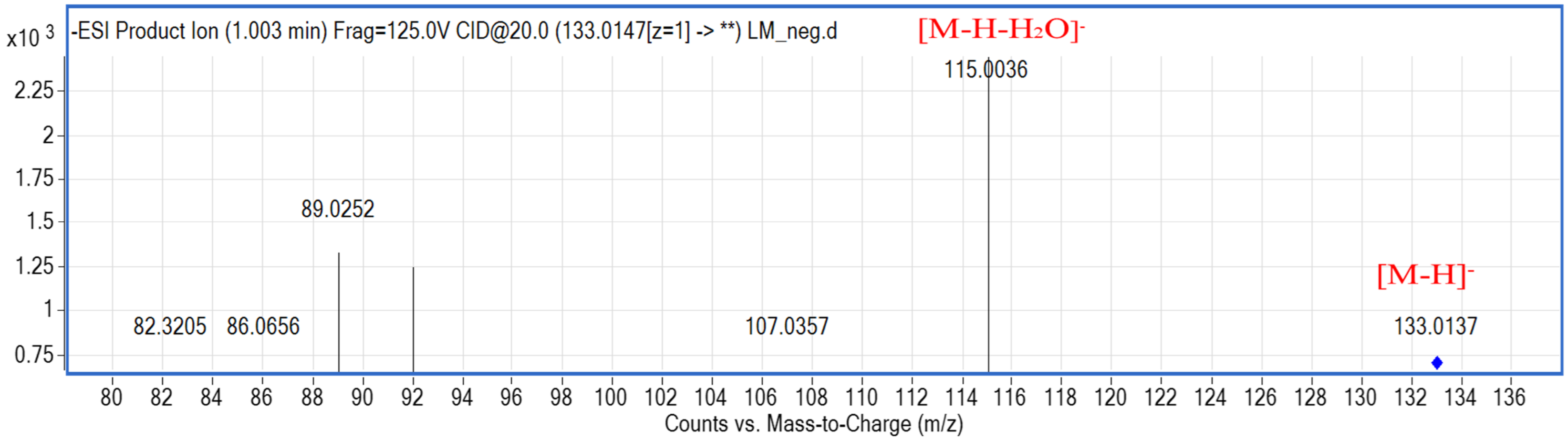

Figure S15: Negative mode MS/MS spectrum of malic acid.

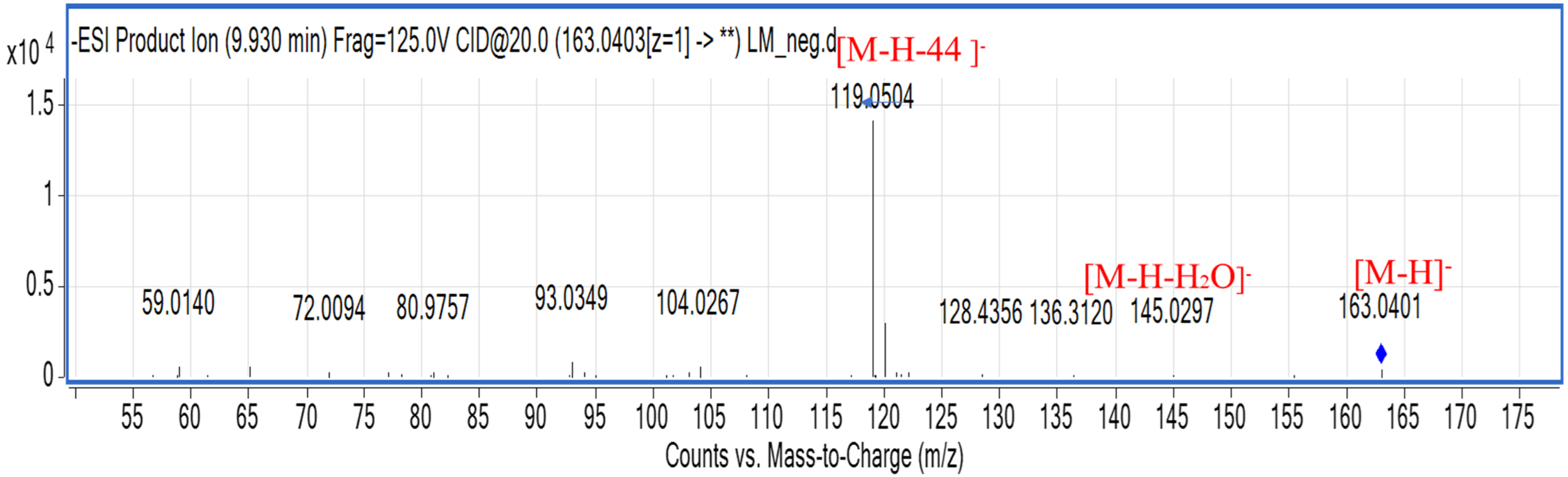

Figure S16: Negative mode MS/MS spectrum of *P*-hydroxy benzoic acid.

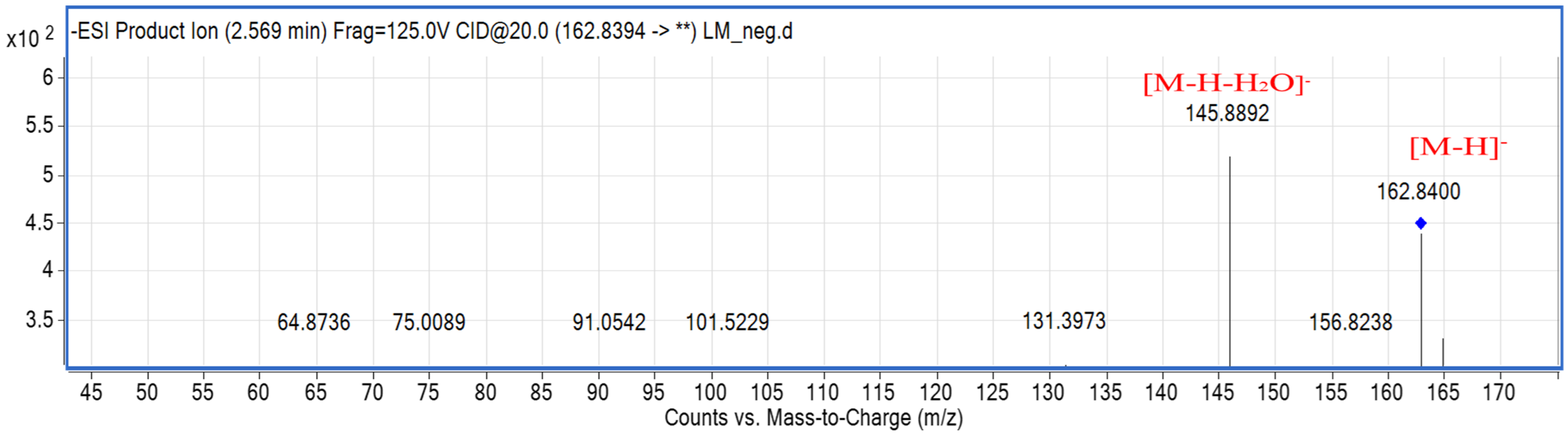

Figure S17: Negative mode MS/MS spectrum of *P*-coumaric acid.

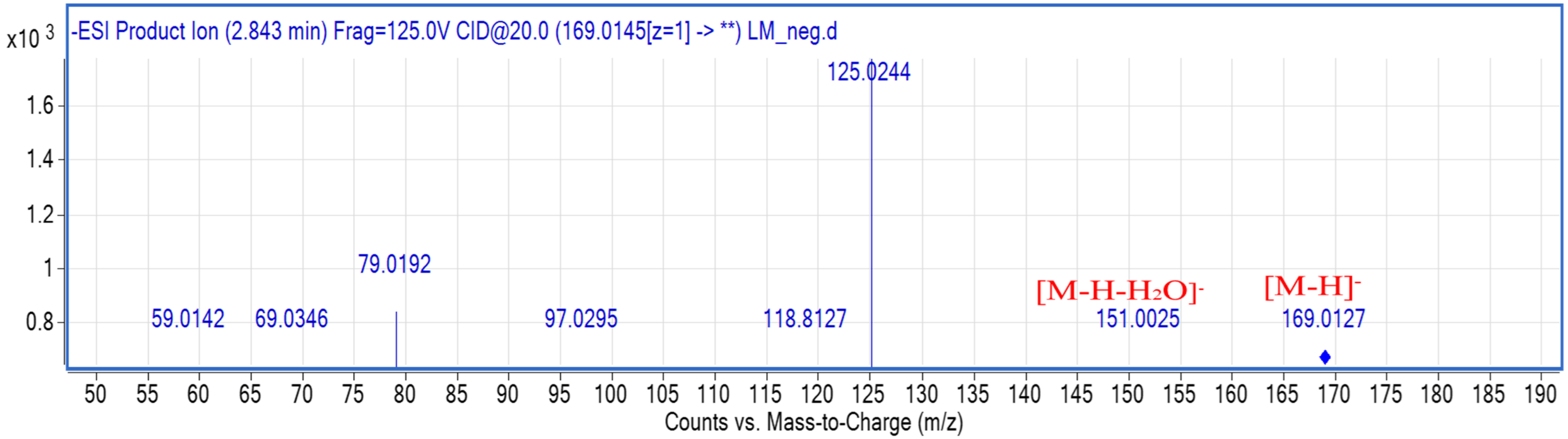

Figure S18: Negative mode MS/MS spectrum of gallic acid.

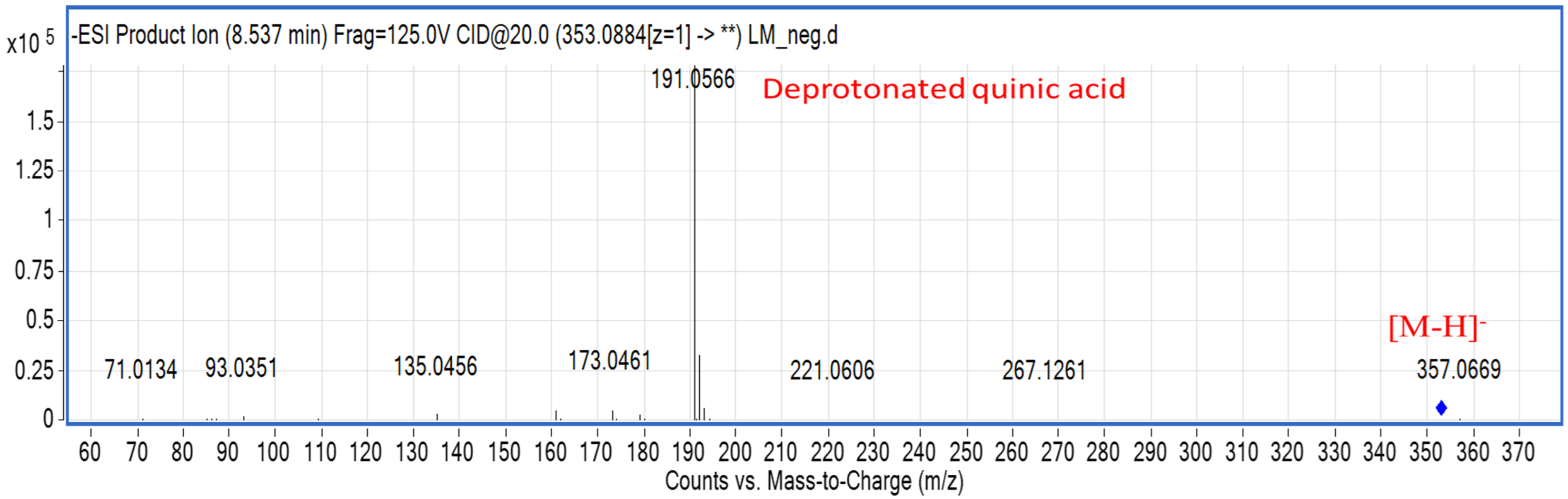

Figure S19: Negative mode MS/MS spectrum of chlorogenic acid.

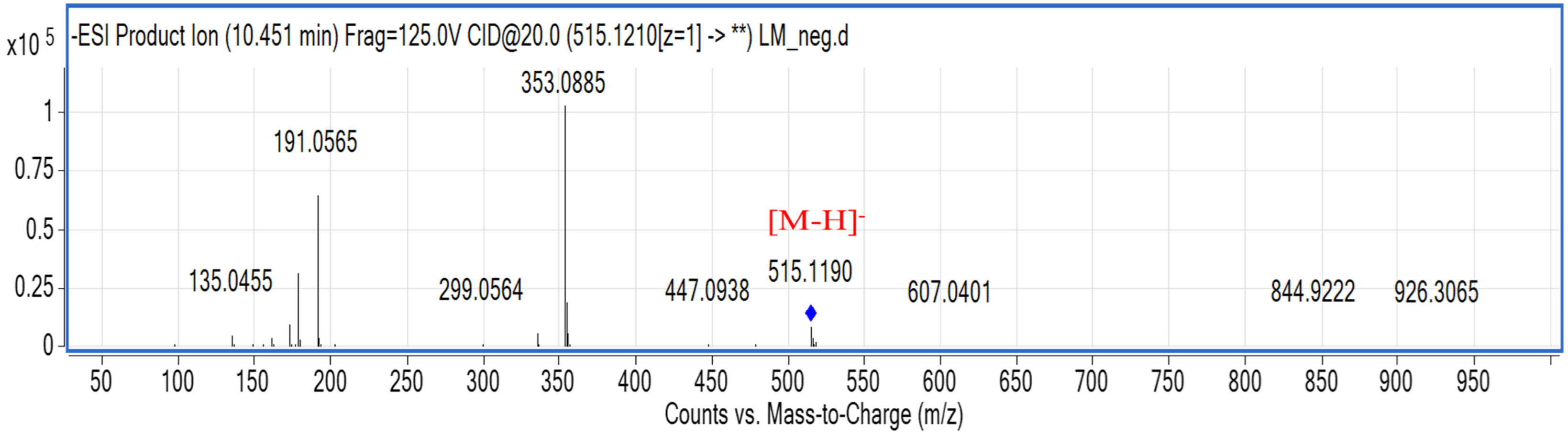

Figure S20: Negative mode MS/MS spectrum of dicaffeoylquinic acid.

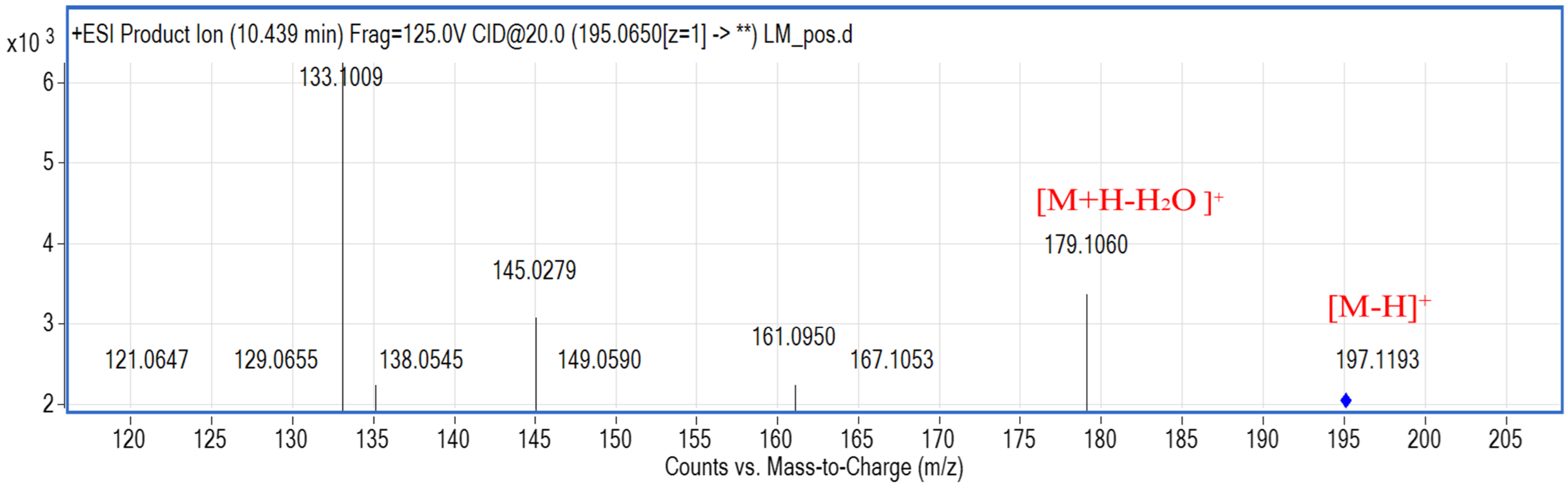

Figure S21: Negative mode MS/MS spectrum of ferulic acid.

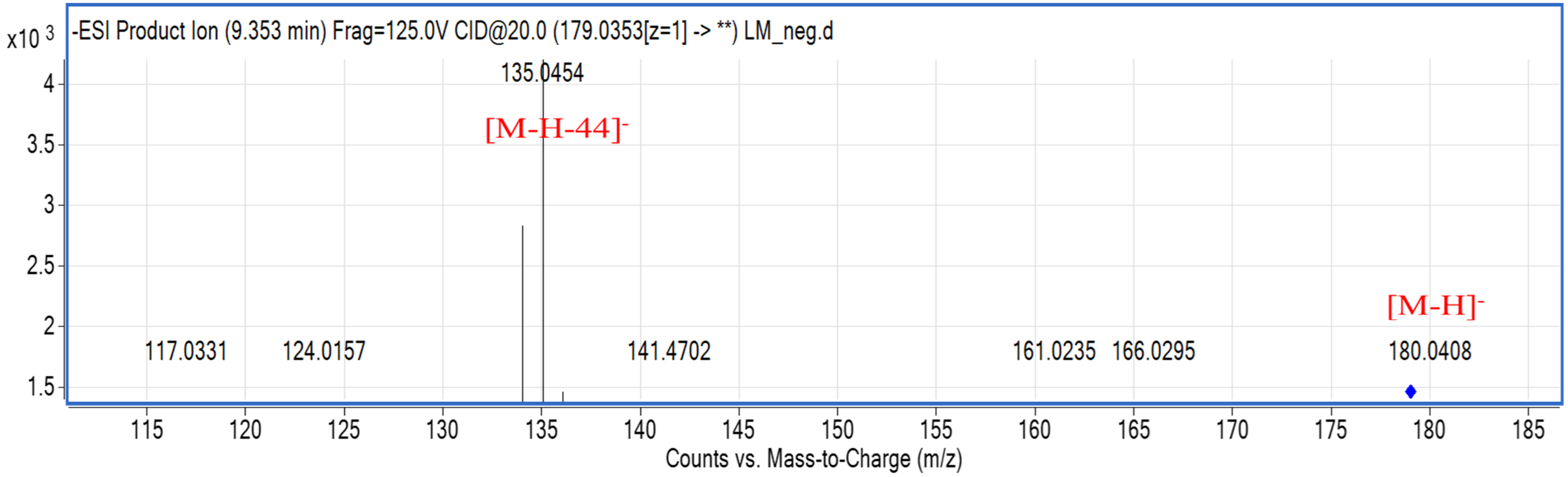

Figure S22: Negative mode MS/MS spectrum of caffeic acid.

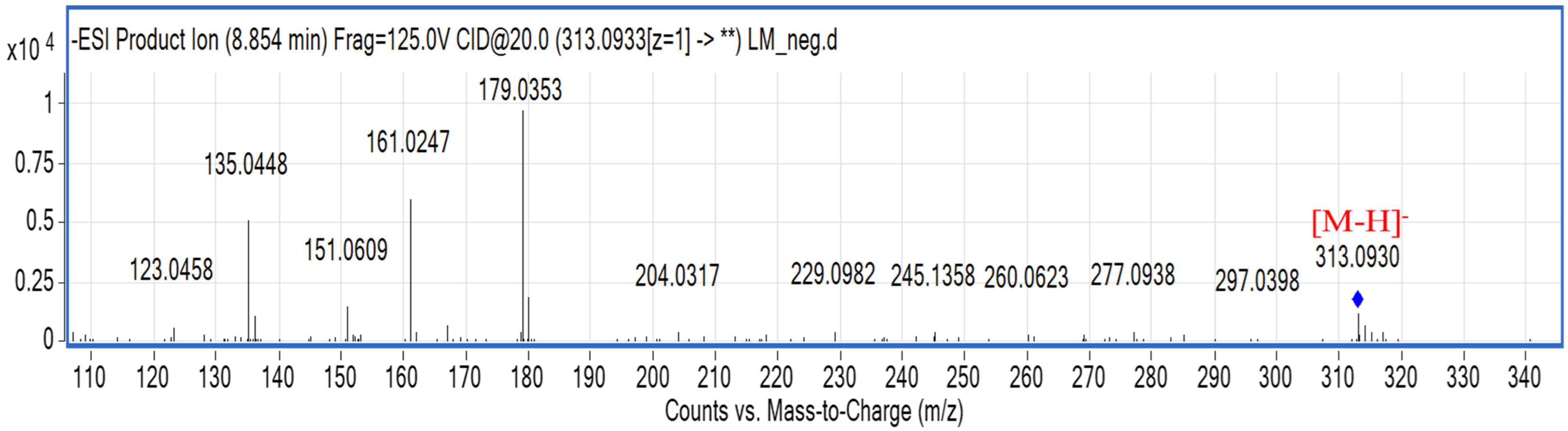

Figure S23: Negative mode MS/MS spectrum of caffeic acid derivative.

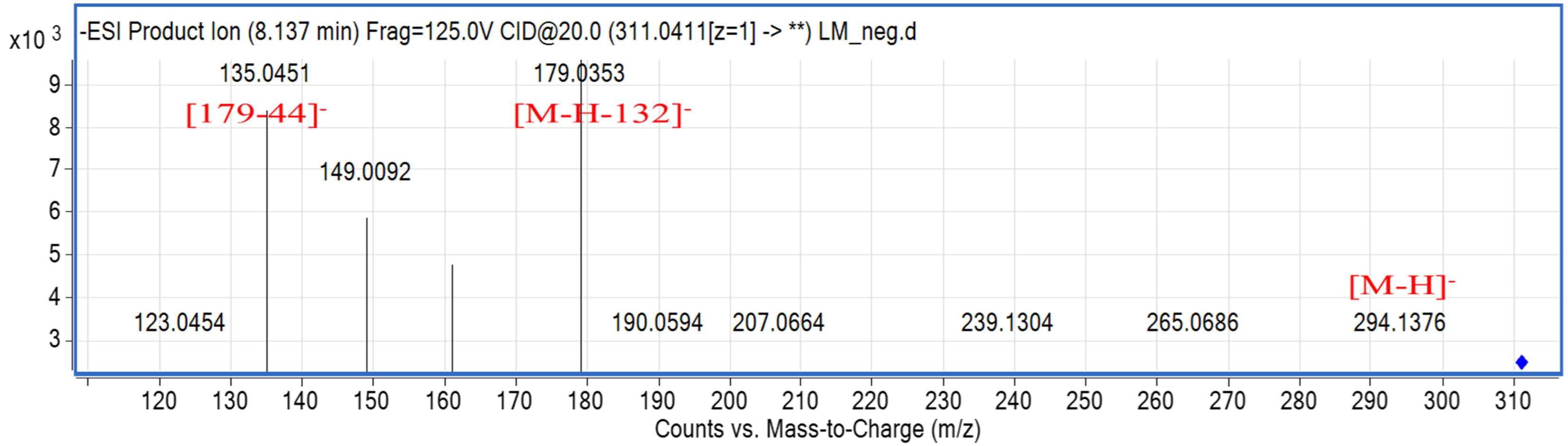

Figure S24: Negative mode MS/MS spectrum of caffeoyl tartaric acid.

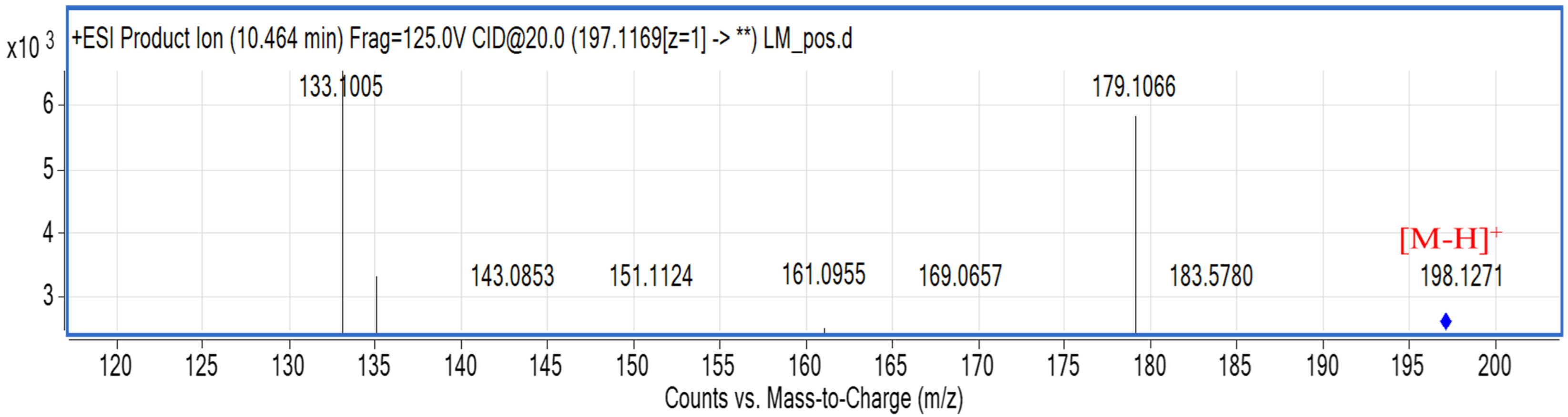

Figure S25: Negative mode MS/MS spectrum of unknown phenolic acid.

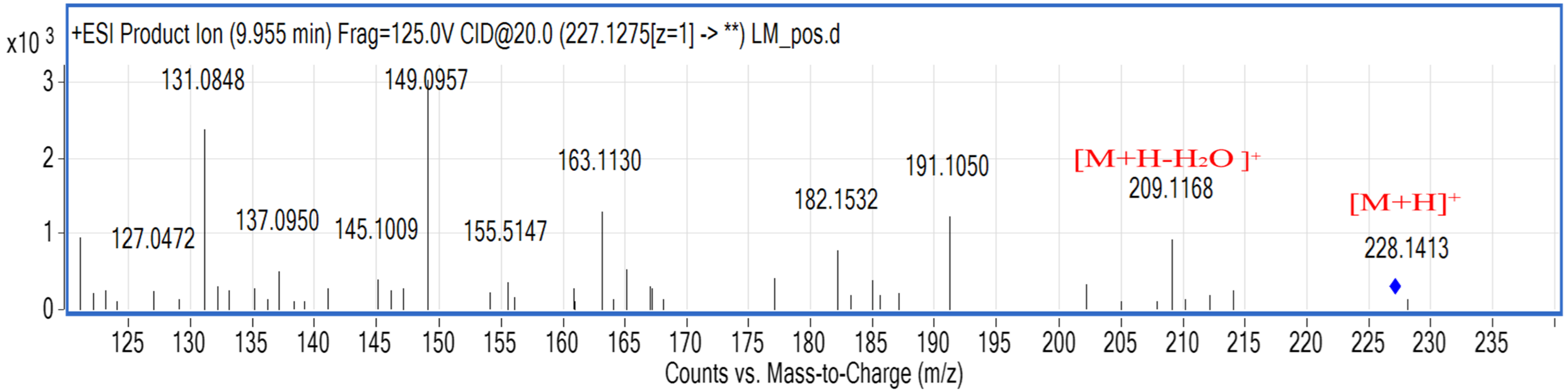

Figure S26: Negative mode MS/MS spectrum of hydroxy jasmonic acid.

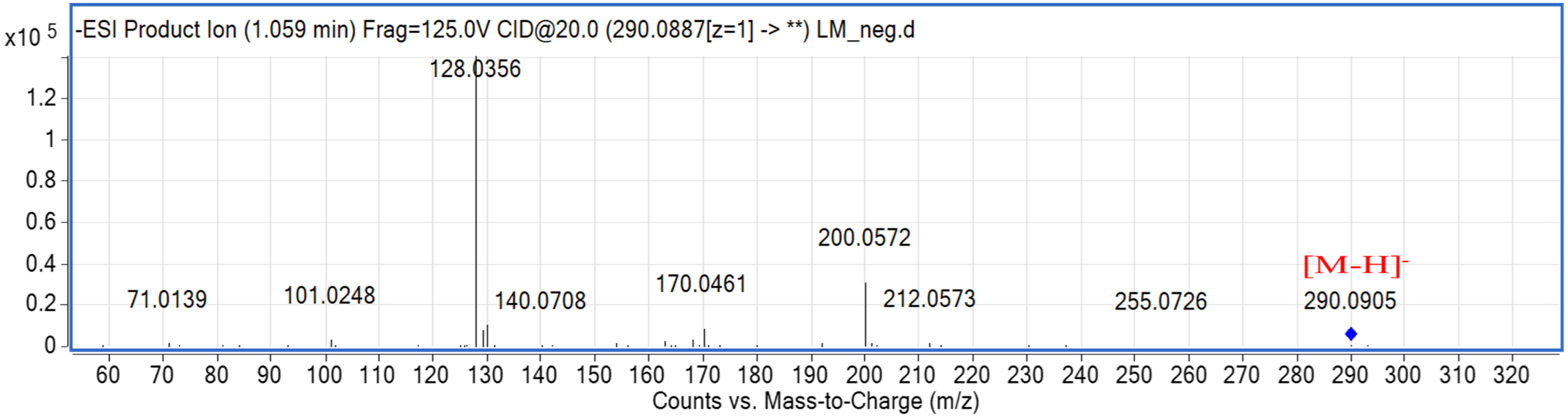

Figure S27: Negative mode MS/MS spectrum of deoxy-dehydro-*N*-acetylneuraminic acid.

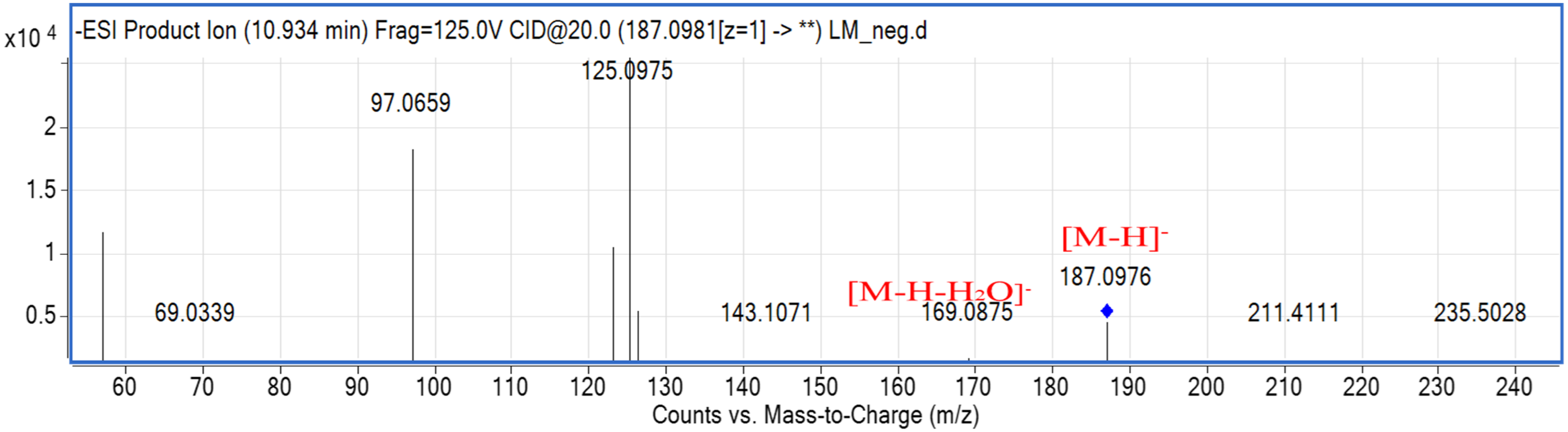

Figure S28: Negative mode MS/MS spectrum of nonanedioic acid.

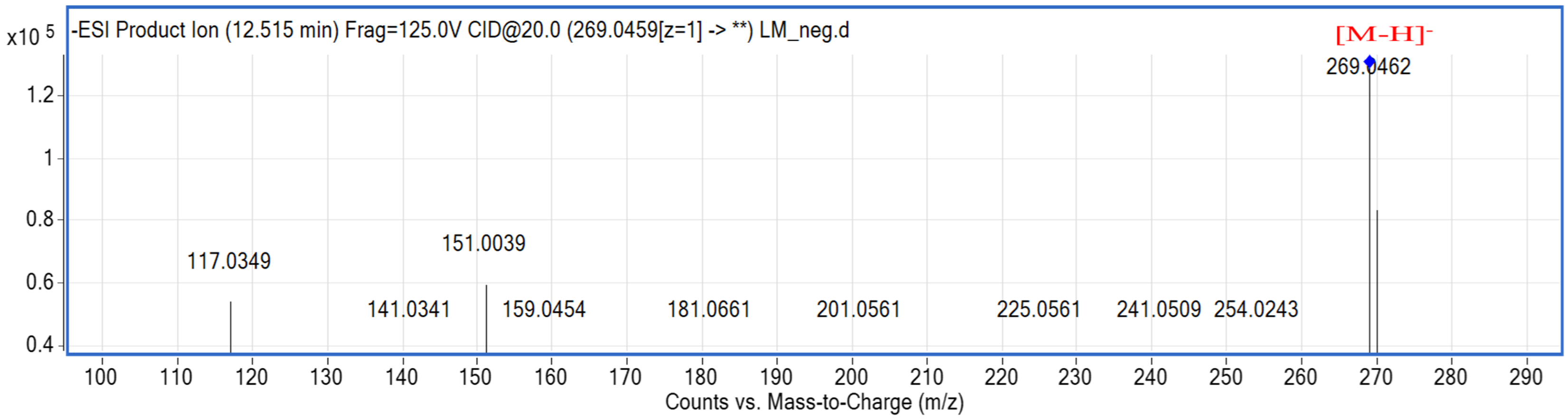

Figure S29: Negative mode MS/MS spectrum of apigenin.

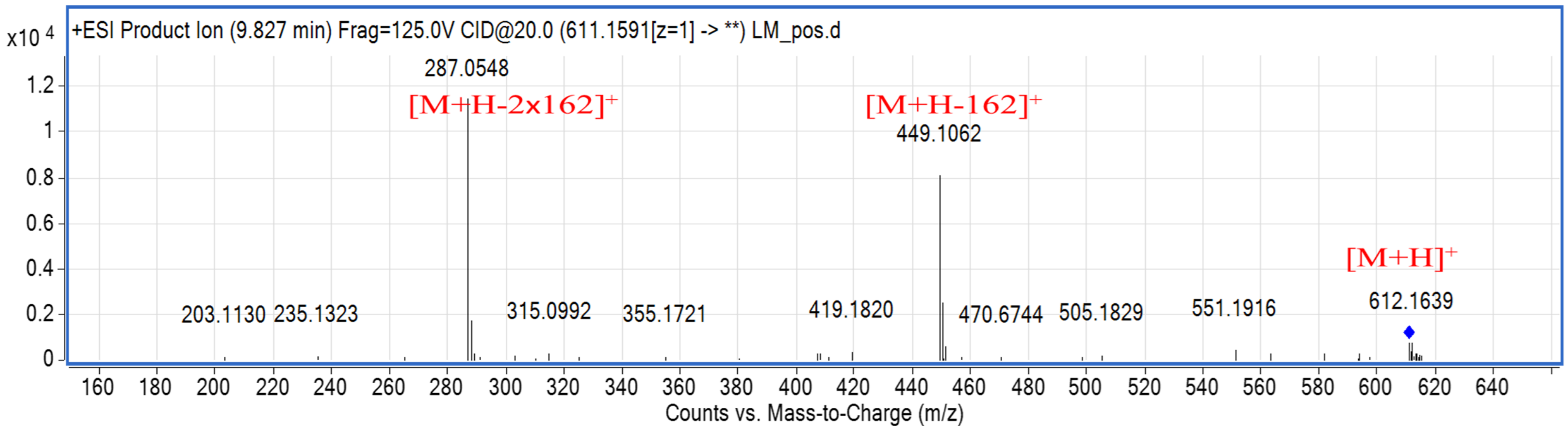

Figure S30: Positive mode MS/MS spectrum of luteolin-*O*-dihexoside.

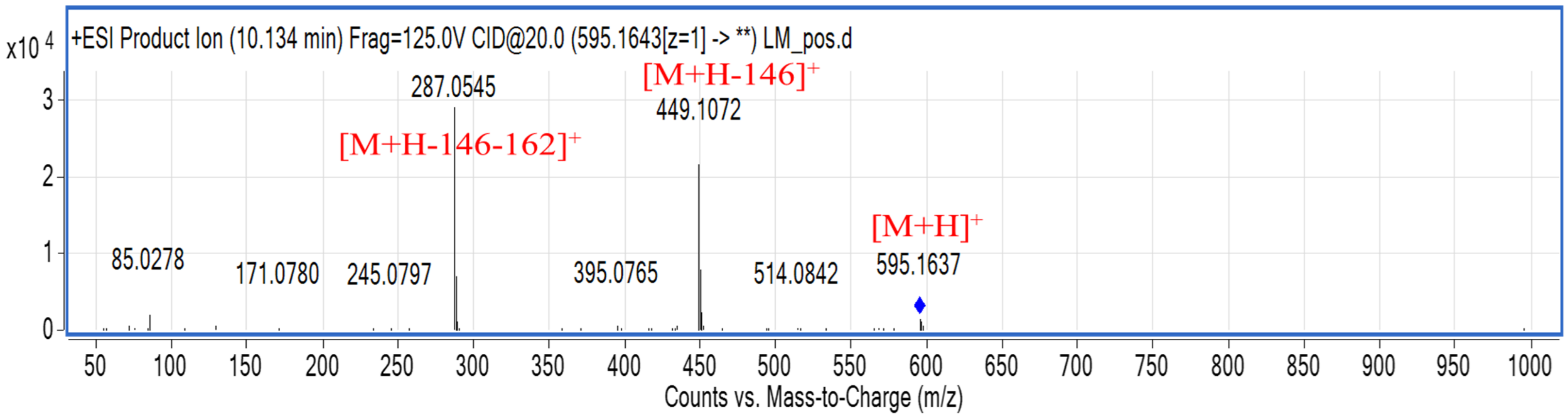

Figure S31: Positive mode MS/MS spectrum of luteolin-*O*-rutinoside.

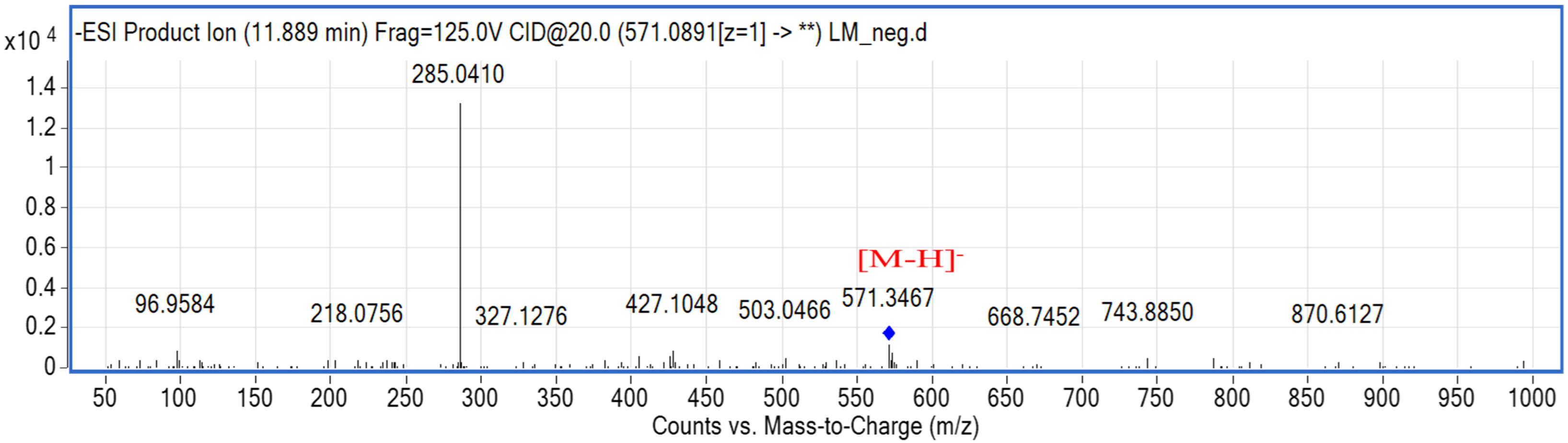

Figure S32: Negative mode MS/MS spectrum of unknown luteolin derivative.

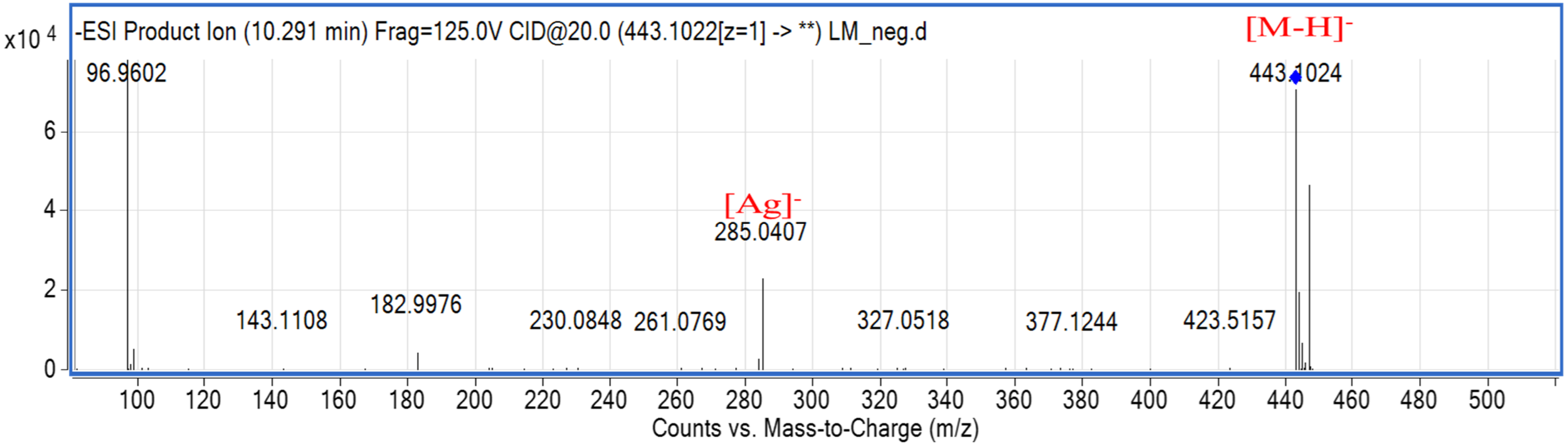

Figure S33: Negative mode MS/MS spectrum of unknown luteolin derivative.

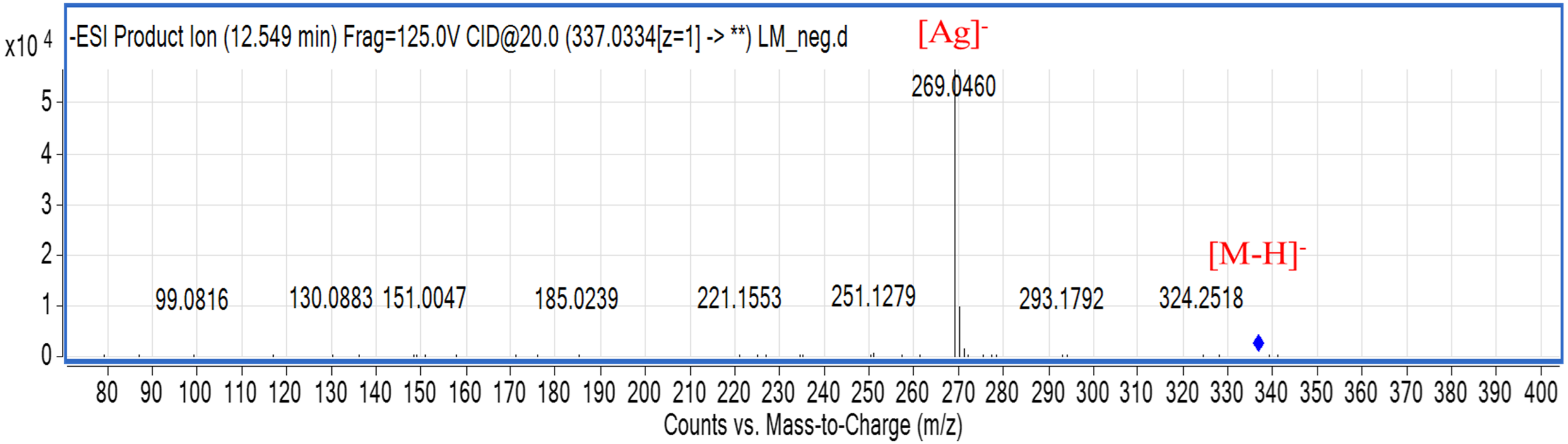

Figure S34: Negative mode MS/MS spectrum of unknown apigenin derivative.

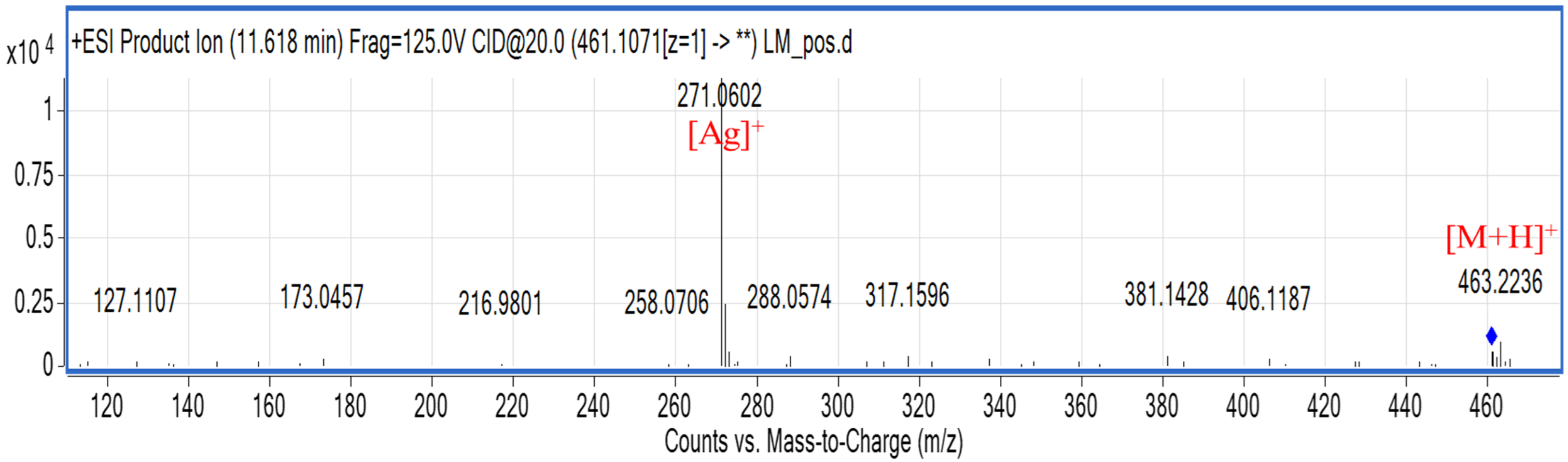

Figure S35: Positive mode MS/MS spectrum of unknown apigenin derivative.

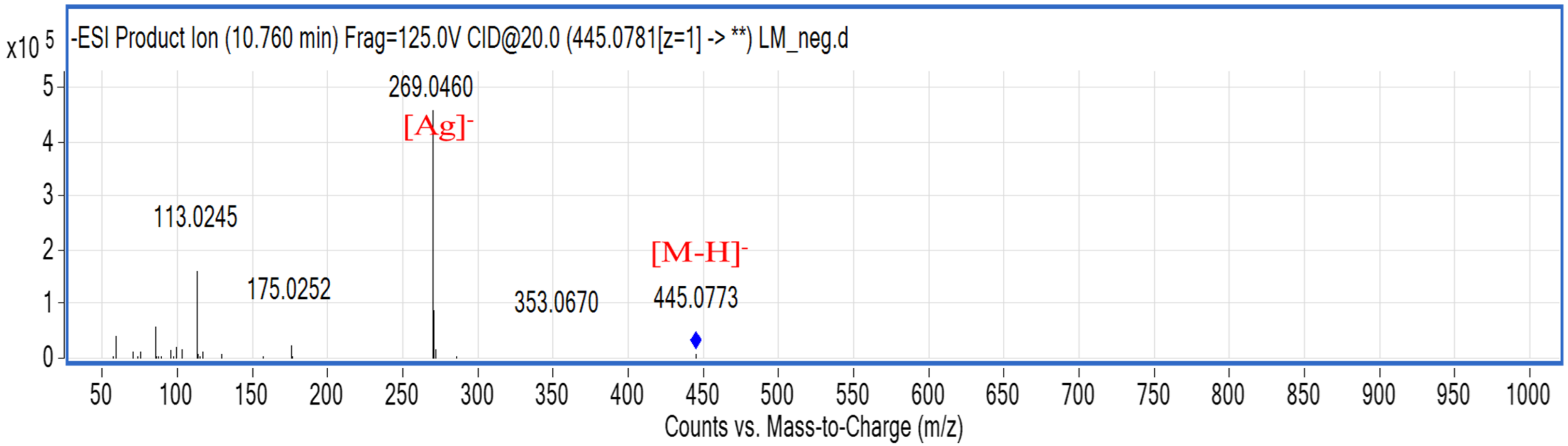

Figure S36: Negative mode MS/MS spectrum of unknown apigenin derivative.

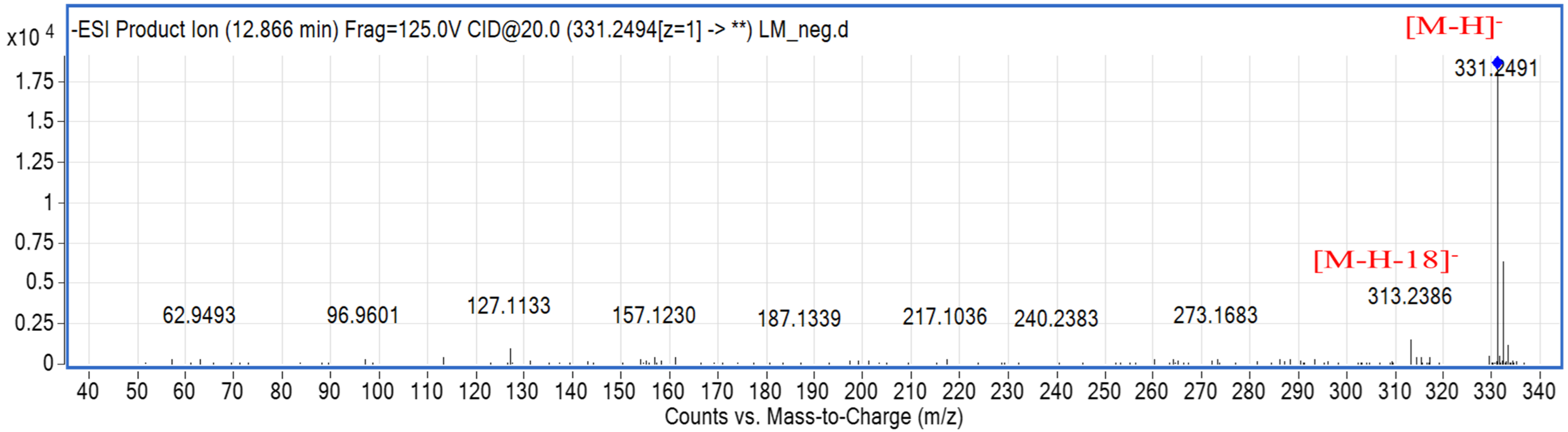

Figure S37: Negative mode MS/MS spectrum of trihydroxy octadecanoic acid.

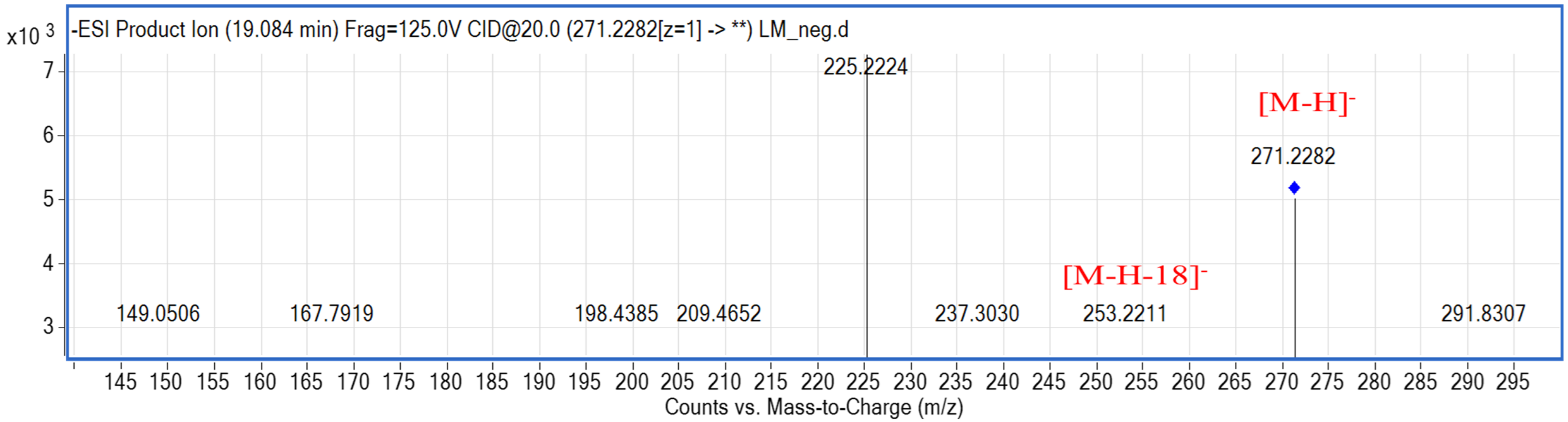

Figure S38: Negative mode MS/MS spectrum of hydroxy palmitic acid.

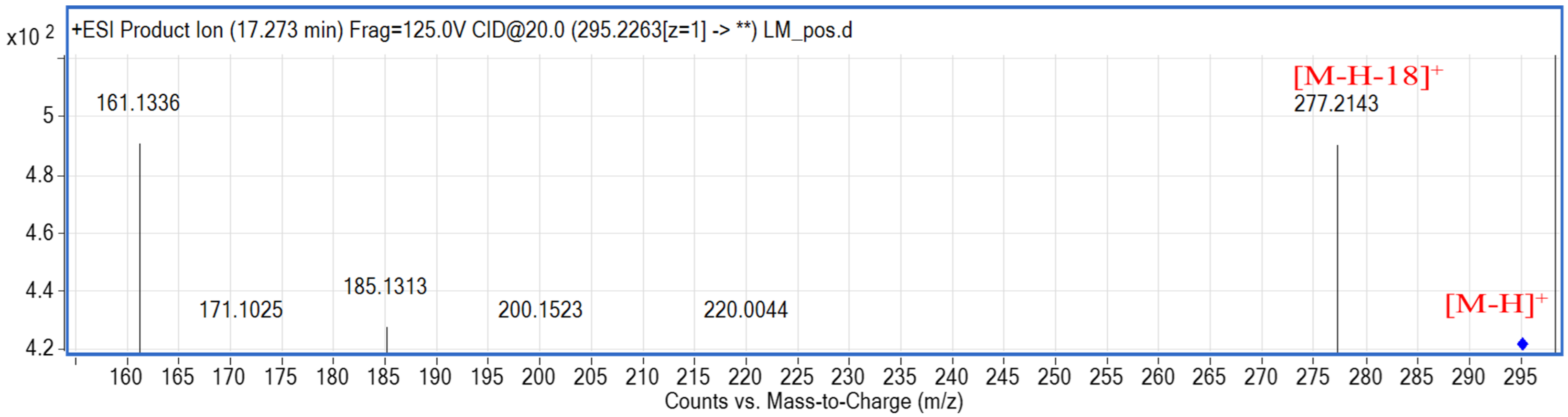

Figure S39: Positive mode MS/MS spectrum of hydroxy linoleic acid.

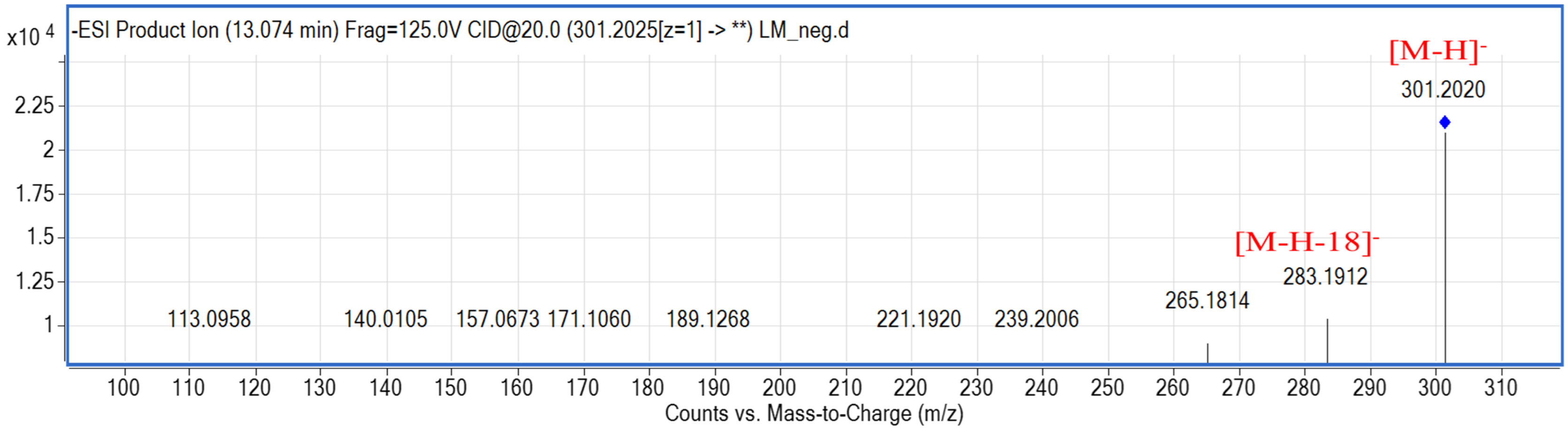

Figure S40: Negative mode MS/MS spectrum of hydroxy hexadecanedioic.

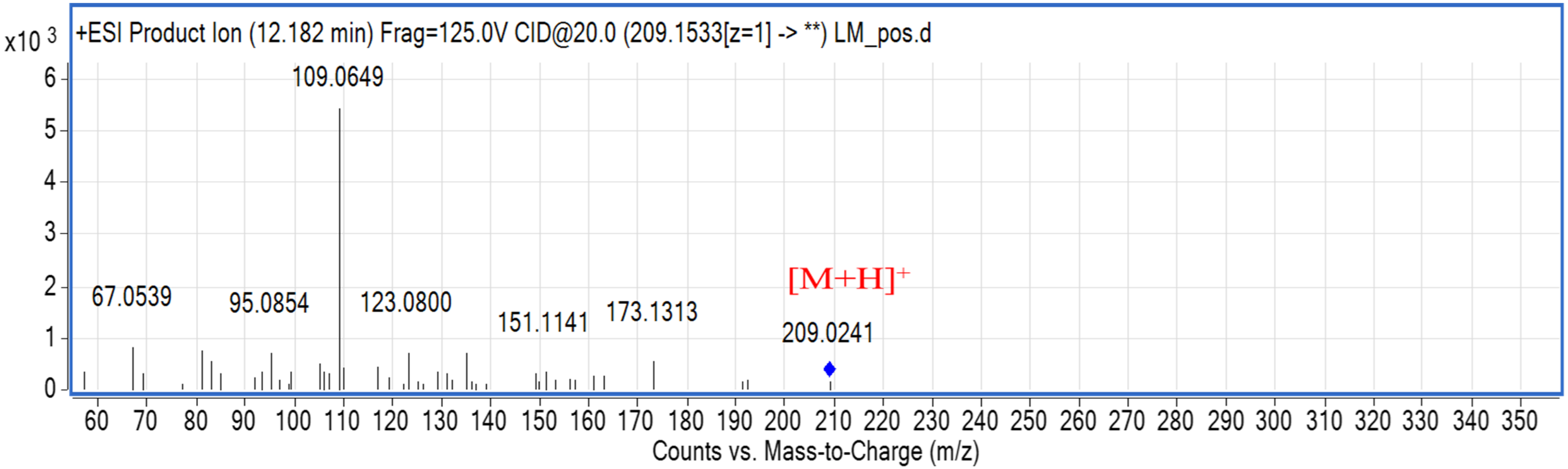

Figure S41: Negative mode MS/MS spectrum of tridecatrienoic acid.

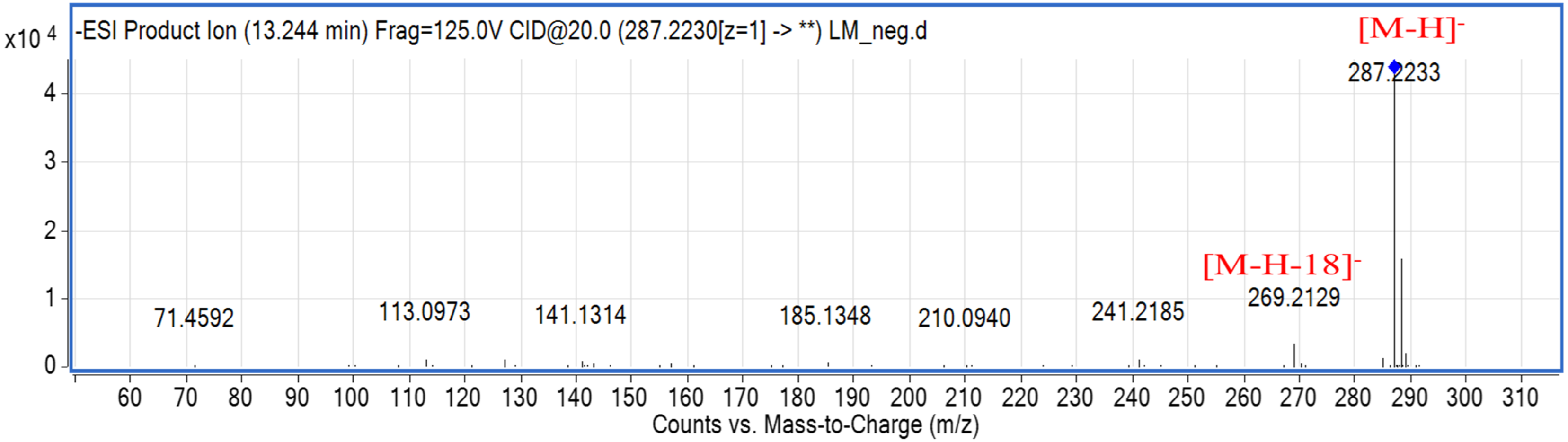

Figure S42: Negative mode MS/MS spectrum of dihydroxy hexadecanoic.

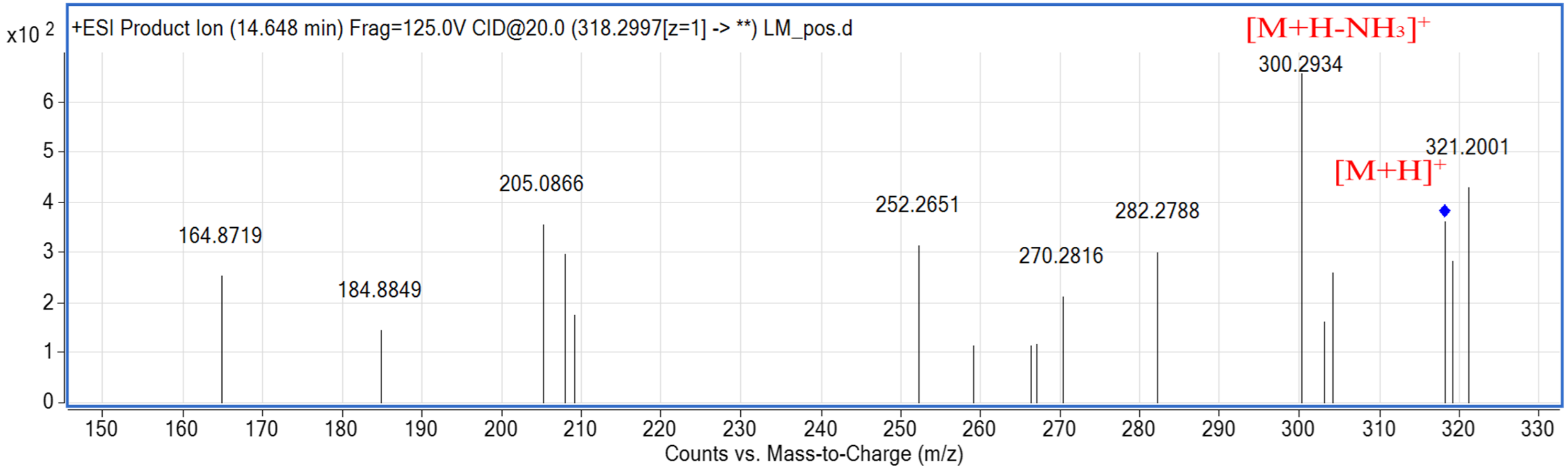

Figure S43: Positive mode MS/MS spectrum of amino octadecanetriol.

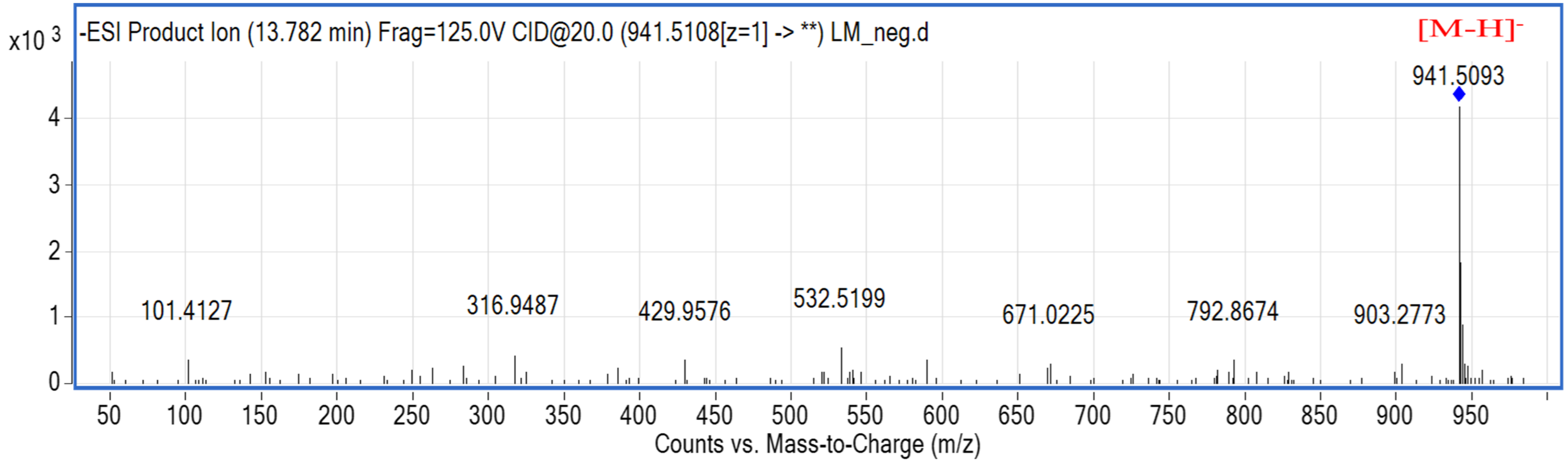

Figure S44: Negative mode MS/MS spectrum of phosphatidylinositol phosphate.
